# Genetic diversity and population structure of U.S. Suffolk sheep participating in the national sheep improvement program

**DOI:** 10.1186/s12711-025-01027-4

**Published:** 2026-01-14

**Authors:** Carrie S. Wilson, Brenda M. Murdoch, Luiz F. Brito, J. Bret Taylor, Artur O. Rocha, Brad A. Freking, Thomas W. Murphy, Ronald M. Lewis

**Affiliations:** 1 Range Sheep Production Efficiency Research Unit, USDA, ARS, Dubois, ID 83423 USA; 2 Department of Animal, Veterinary and Food Sciences, University of Idaho, Moscow, ID 83844 USA; 3 Department of Animal Sciences, Purdue University, West Lafayette, IN 47907 USA; 4 USDA, ARS, Roman L. Hruska U.S. Meat Animal Research Center, Clay Center, NE 68933 USA; 5 Department of Animal Science, University of Nebraska-Lincoln, Lincoln, NE 68583 USA

## Abstract

**Background:**

The Suffolk is the primary terminal sire breed in the U.S. As a breed that participates in the National Sheep Improvement Program (NSIP), Suffolk breeders are attempting to accumulate enough genomic information to provide genomic-enhanced estimated breeding values as part of the national genetic evaluations. The effective implementation of genomic selection and management of genetic diversity in the breed require a comprehensive assessment of current genetic diversity and population structure. Therefore, the primary objective of this study is to assess the genetic diversity and population structure present in U.S. Suffolk sheep included in the NSIP using both pedigree- and genomic-based methods. A secondary objective is to compare the levels of genomic diversity of U.S. Suffolk to those from other selected countries.

**Results:**

Based on pedigree (*n* = 75,161) analyses, the generation interval was 2.8 years, and the effective number of founders and ancestors were 504 and 300, respectively. Effective population size ranged from 28 to 194 based on pedigree-based measures and 75 to 85 based on genomic-based metrics. When the mean inbreeding was compared for the 1,878 genotyped animals (GGP Ovine 50 K BeadChip) that passed quality control, pedigree-based inbreeding; and, inbreeding based on heterozygosity, runs of homozygosity, diagonal of the genomic relationship matrix, and homozygous-by-descent segments were 4.8%, 3.3%, 4.6%, 3.3%, and 3.4%, respectively. Of the 16 flocks with genotyped animals, four had fixation index values that exceeded 0.10, but the model-based population structure showed admixture across all flocks. For the principal component analysis and the model-based population structure with international genomic datasets, the U.S. Suffolks were distinct, the United Kingdom Suffolks were placed in-between but distinct from the other countries, and the Australian, Irish, and New Zealand Suffolks were grouped together.

**Conclusions:**

The current level of genetic diversity and population structure was quantified for the U.S. Suffolk breed. While the rate of inbreeding was at an acceptable level, the effective population size was modest, indicating that monitoring of genetic diversity and strategic mating of less related animals in the breed should continue. As the sheep industry moves forward, regular assessments of genetic diversity and population structure are needed.

**Supplementary Information:**

The online version contains supplementary material available at 10.1186/s12711-025-01027-4.

## Background

The underlying genetic composition of a breed both distinguishes it from others and provides a means from which to make genetic change in the population. While an understanding of the genetic diversity and population structure of a breed is needed, it becomes more critical as the U.S. sheep industry moves toward implementation of genomic selection (GS) on a national scale. Although GS allows breeders to make more accurate breeding decisions, unintended consequences of GS are possible, including reduced genetic diversity and negative impacts on secondary traits [1–3]. A baseline assessment prior to implementation of GS is warranted to which future assessments of genetic diversity can be compared. In addition, genetic diversity estimates are useful for predicting expected accuracy of genomic-enhanced estimated breeding values based on trait heritability, size of the reference populations, and the extent of linkage disequilibrium (LD) between the markers and the causal variant [4].

Of the major U.S. sheep breeds participating in national genetic evaluations through the National Sheep Improvement Program (NSIP) [5], genetic diversity studies have been conducted for Katahdin [6, 7] and Polypay [8]. Such an investigation was also conducted in Suffolk sheep but included a limited number of genotyped animals from NSIP flocks [9]. In the present study, we applied similar methods to a much larger number of genotyped NSIP Suffolk sheep. While the exact number of U.S. Suffolk flocks is unknown, the NSIP Suffolk participating flocks are expected to represent less than 50% of the total number of flocks.

Suffolk is an important terminal sire breed in the U.S. and globally. It was developed in the lowlands of the United Kingdom in 1810 and its breed society was formed in 1887 [10]. The breed was formed by crossing Southdown rams with Norfolk Horned ewes, with the resulting offspring being considered superior to either parent breed [11]. The first Suffolks were brought to the U.S. in 1888 [11]. The breed is characterized by a wool-free black head and legs, with a white fleece that ranges from 26 to 33 microns, weighs 1.8 to 3.6 kg (ewe) with a 50 to 60% clean fleece yield, and a 5 to 9 cm staple length [12]. Their popularity as a terminal sire breed is due to rapid early growth, heavy muscling, and improved feed efficiency [11, 13, 14].

The main objective of this study is to assess the genetic diversity and population structure of U.S. Suffolk sheep included in NSIP using both pedigree- and genomic-based methods. Pedigree-based methods allow animals from the entire population to be included, tracing back many generations. Genomic-based methods allow genetic diversity to be assessed on a much finer scale, but such data is rarely available for the entire population. Because the Suffolk breed is utilized throughout the world, an additional objective was to compare the U.S. NSIP Suffolk population to those from other countries using publicly available genomic data.

## Methods

### Pedigree analyses

The pedigree was obtained for all Suffolk sheep participating in NSIP, which consisted of 128 flocks. The pedigree records were traced back until all ancestors were unknown using the Animal Breeders Toolkit [15]. The final pedigree included 75,161 animals with 3450 sires and 16,812 dams. The number of progeny ranged from 1 to 355 for sires and 1 to 26 for dams. A maximum of 24 generations were present in the available pedigree data.

### Pedigree subgroups

The ENDOG software [16] was used for the pedigree analyses, and considered the full pedigree and four key subgroups. The subgroups were designed to provide information about recent generations. As with the full population, analyses from all subgroups included relationships that traced back until all ancestors were unknown. Subgroup 1 (SG 1) included animals from the most recent generation, defined as those born from 2021 to 2023. Subgroup 2 (SG 2) included animals from SG 1 with a minimum of a 4-generation known pedigree. To allow for direct comparisons to the genomic-based analyses, subgroup 3 (SG 3) included available animals genotyped with the GGP Ovine 50 K BeadChip (Neogen, Lincoln, NE, USA) that met quality control standards. To evaluate the animals contributing to the most recent generation, subgroup 4 (SG 4) included sires of animals in SG 1. The number of animals included in each subgroup were 5159, 4065, 1878, and 216, for SG 1, SG 2, SG 3, and SG 4, respectively.

### Generations

The mean generation interval was computed using the four-path method, with the average of the pathways between the sire-son, sire-daughter, dam-son, and dam-daughter calculated [17, 18]. For the full pedigree and each subgroup, the mean maximum number of generations, mean number of complete generations, the equivalent complete generations, and the pedigree completeness index (PCI) were determined. The mean number of maximum generations was defined based on the number of generations between an animal and its furthest ancestor. Mean complete generations was the average number of generations between an animal and its furthest generation where all ancestors were known [16]. The equivalent complete generations were computed as the sum of (1/2)^n^, where n was the number of generations between the individual and each known ancestor [19]. PCI was computed for each generation based on the completeness of the pedigree in previous generations [20].

### Pedigree-based inbreeding

Inbreeding coefficients were computed with the method of Meuwissen and Luo [21] using the ENDOG software [16]. The change in inbreeding over time, ΔF, was computed. To evaluate whether the ΔF was increasing at a significantly greater rate, a linear model was fit in the R software to assess if the regression slope of ΔF on generation (*n* = 2.8 years) was significantly different from 0 [22]. The average relatedness (AR) was the probability that a randomly chosen allele from the whole population in the pedigree belonged to a given animal. It was used to predict the long-term inbreeding of the population by accounting for both inbreeding and coancestry coefficients [16]. The AR was twice the mean coancestry between an animal and all animals in the pedigree, including itself [23].

### Ancestors and founders

The evaluation of ancestors and founders in the pedigree included the effective number of ancestors (*f*_*a*_), the effective number of founders (*f*_*e*_), the ratio of *f*_*e*_ to *f*_*a*_, the marginal contributions of ancestors, the number of ancestors contributing to 50% of the genetic diversity of the population, and the genetic conservation index (GCI). The *f*_*a*_ determines the minimum number of ancestors that explain the genetic diversity of the population. The ancestors may be, but were not required to have been, from the founder population and were chosen based on their expected genetic contribution [24]. The *f*_*e*_ was defined as the number of equally contributing founders that would produce the same genetic diversity as observed in the population [16, 25]. The ratio of *f*_*e*_ to *f*_*a*_ was compared to identify genetic bottlenecks, where larger values are associated with more narrow bottlenecks [24]. The marginal contributions of ancestors represent the genetic contribution made by an ancestor that is not explained by previously selected ancestors. Accumulated marginal contributions by number of ancestors were plotted. The top 10 marginal contributors were identified and described. The number of ancestors contributing to 50% of the genetic diversity of the population was calculated. This value allows an assessment of whether there are few highly influential ancestors or many lowly influential ancestors contributing to the current gene pool.

The GCI quantifies the proportion of genes from each founder in the pedigree of each animal such that genetic diversity is maximized when each founder contributes equally to each animal. Computationally, the GCI for an animal is the effective number of founders in its pedigree from 1/$\:{P}_{i}^{2}$, where $\:{P}_{i}$ is the proportion of genes of founder animal *i* in the pedigree. The maximum GCI value is equal to the number of founder ancestors in the breed. Therefore, the higher an animal’s GCI value, the more genetic diversity it is expected to have. A limitation of the use of GCI is the value becomes more informative as the depth of pedigree increases; thus, comparisons across different pedigree depths cannot be made [26].

### Effective population size (Ne)

Seven measures of N_e_ were computed using the ENDOG software [16]. The methods included the individual increase in inbreeding. Additional estimates of N_e_ were based on the increase in inbreeding over maximum generations, complete generations, and equivalent complete generations traced [27, 28]. The regression of the inbreeding coefficients on year of birth N_e_ also was obtained as described by Gutiérrez et al. [29] as was the log regression of (1–F) on generation number as described by Pérez-Enciso [30]. The N_e_ was estimated from the increase in coancestry for all pairs of individuals [31]. Due to computational limitations of calculating the increase in coancestry, animals from SG 1 (1.3 million relationships) were used for the final N_e_ computation instead of the full pedigree (2.8 billion relationships). The full pedigree was used for the remaining N_e_ measures.

### Pedigree-based population structure

The population structure was examined for the full pedigree by flock using both Wright’s fixation index (F_ST_) [32–34] and Nei’s minimum distance [35]. Wright’s F_ST_ was computed as:

1 $$\:{F}_{ST}=\:\left(\stackrel{-}{f}-\:\stackrel{\sim}{f}\right)\:/\:\left(1-\:\stackrel{\sim}{f}\right),$$

where $\:\stackrel{-}{f}$ was the average coancestry for the subpopulation (flock), and $\:\stackrel{\sim}{f}$ was the mean coancestry for the entire population. The Nei’s minimum distance (D) was computed as:

2 $$\:{D}_{ij}=\:\left[\left({f}_{ii}+\:{f}_{jj}\right)/2\right]-\:{f}_{ij},$$

where *f*_*ii*_ and *f*_*jj*_ were the average coancestry within populations *i* and *j*, respectively, and *f*_*ij*_ was the average coancestry between the two populations.

### Genomic analyses

#### Quality control

A total of 1,909 sheep were genotyped with the GGP Ovine 50 K BeadChip, which included 51,867 markers. Quality control procedures were performed using the PLINK software (version 1.90) [36]. After filtering for animals with unknown parentage (*n* = 9) and animal call rate < 0.90 (*n* = 22), genotypic data from 1,878 animals remained for subsequent analyses. The remaining animals included 1,195 females and 683 males with birth years from 2010 to 2024. There were 16 NSIP-participating flocks represented with an average of 117 (SD = 76) genotyped animals per flock with a range of 12–238. Only autosomal single nucleotide polymorphisms (SNP) with call rate > 0.90 were retained for subsequent analyses. Flanking SNP in LD equating to an r^2^ value of more than 0.5 were removed [37, 38]. There were 35,543 markers remaining in the “full SNP” dataset, which was used for determining minor allele frequency (MAF) categories, runs of homozygosity, and homozygous-by-descent (HBD) segments, where inclusion of fixed alleles was an integral part of the analyses. For most analyses, the markers were further filtered for fixed and rare alleles (MAF < 0.01), leaving 34,243 markers in the “reduced SNP” dataset. These analyses included genomic-based N_e_, heterozygosity, F_ST_, principal component analysis (PCA), and model-based population structure. For the LD analyses, markers were not filtered based on LD; only markers with MAF < 0.01 were removed, leaving 44,094 markers in the “LD SNP” dataset.

#### Genomic diversity and inbreeding

One genomic-based N_e_ estimate was computed for the reduced SNP dataset with the NeEstimator software package [39] using the LD method [39, 40] with jackknife confidence intervals as described by Waples and Do [40]. Although the reduced SNP dataset was filtered to remove markers in LD and this may appear to be in conflict with the LD method of NeEstimator, the software authors reported that there is little value of including more than 10,000 markers in the analysis. Consequently, removing markers in LD or additional thinning of the markers would not be expected to influence the results. An additional genomic-based N_e_ estimate was computed using the reduced SNP dataset with the GONE software package [41]. The MAF categories were delineated in increments of 0.05 using the PLINK software with the full SNP dataset [8, 42]. Expected (H_E_) and observed (H_O_) heterozygosity were computed using the reduced SNP dataset in PLINK. Then, the individual inbreeding coefficient based on heterozygosity, F_IND_, was computed:

3 $$\:{F}_{IND}=\left({H}_{E}-{H}_{0}\right)/{H}_{E}.$$

Runs of homozygosity (ROH) were detected with a sliding window of 50 SNP with a minimum length of 1000 kb (equivalent to ~ 30 SNP), a maximum distance allowed between SNP within an ROH of 250 kb, a minimum of 30 SNP, allowing for one missing SNP, and allowing a maximum of one heterozygous SNP (allowing a genotyping error rate of 3%) within the defined window with a window threshold of 0.05 using the DetectRUNS R package [43] with the full SNP dataset. Parameters for ROH detection were determined using recommendations by Biscarini et al. [42] and other livestock studies using medium density arrays including Dzomba et al. [44]. The ROH class categories were: 1 to 6, > 6 to 12, > 12 to 24, and > 24 Mb pairs. The number of ROH segments per animal was calculated. The inbreeding based on ROH (F_ROH_) was computed as the total length of the autosomal genome covered by ROH divided by the total length of the genome covered by SNP (after quality control). The F_ROH_ was computed by ROH class category to evaluate historic versus recent inbreeding.

Homozygous-by-descent (HBD) segments were detected using the full SNP dataset using the RZooRoH R package [45, 46]. Identification of HBD and non-HBD segments are based on the hidden Markov model, which estimates the probability of autozygosity along an individual’s genome. The HBD analyses are less sensitive to parameter definitions than ROH methods. The HBD inbreeding coefficient, F_HBD_, is the proportion of the genome that is estimated to be autozygous.

The genomic relationship matrix (GRM) was computed using VanRaden’s first method [47] with the GCTA software package [48] with the reduced SNP dataset. Then, the genomic inbreeding coefficient based on the GRM (F_GRM_) was computed using the R software as F_i_ = G_ii_ − 1, where F_i_ is the inbreeding coefficient for individual i and G_ii_ is the diagonal element of the GRM for individual i [47]. Pearson correlation coefficients were computed between the measures of inbreeding for the genotyped animals, which included pedigree-based F, F_IND_, F_ROH_, F_ROH_ categories, F_GRM_, and F_HBD_.

The LD was computed using the r^2^ method in PLINK [49] using the LD SNP dataset. The SNP pairs were assigned to bins based on pairwise marker distance, and the average of each bin was plotted to illustrate the LD decay with increasing physical distance [8, 42]. The average distance and LD (r^2^) between adjacent SNP markers were calculated.

#### Genomic-based population structure

Genetic differences within the population were compared at the flock level and included F_ST_, PCA, and model-based population structure. For the 16 flocks with genotyped animals, the F_ST_ was computed using the reduced SNP dataset with the StAMPP R package [50]. Bootstrapping (*n* = 100) produced 95% confidence intervals around pairwise F_ST_ values.

For the PCA, to avoid overrepresentation of flocks with more genotyped animals, a maximum of 30 animals were included per flock. This was achieved by randomly sampling 30 animals per flock for the 13 flocks with more than 30 genotyped animals for five replicates using the R software. The three flocks with 30 or fewer genotyped animals were included in all replicates. Each replicate included 461 animals. Using the reduced SNP dataset, a distance matrix for each replicate was computed using PLINK by extracting the eigenvectors. Combinations of principal components (PC) 1, 2, and 3 were visualized by flock for each replicate using the R software and the percentage of variation explained by each PC was computed.

The model-based population structure was determined for genotyped animals using the ADMIXTURE software [51] with the reduced SNP dataset. ADMIXTURE used the genotype matrix to identify ancestral populations and then assigned animals proportionally to those populations based on allele frequencies. ADMIXTURE was run for 2 to 20 ancestral populations (K). The general recommendation for determining the number of K is to use the lowest cross-validation error compared to other K values [51]. After 20 K, the cross-validation error was still decreasing. This is far beyond the number of ancestral populations expected for a single breed such as Suffolk. Therefore, the relative decrease in cross-validation error and expectations based on the known biological history of the breed was used to inform the number of K [52]. The CLUMPP program was used to align and merge the replicates of the coancestry coefficient matrix, **Q**, produced by ADMIXTURE [53]. The STRUCTURE PLOT package [54] was used to generate bar plots to visualize each animal by flock.

#### International comparison

Multiple Suffolk genomic datasets were downloaded from the International Sheep Genomics Consortium 1,000 Sheep Genomes Project database (https://www.sheephapmap.org). The datasets included 19 animals from New Zealand (AgResearch OvineHD), 109 from Australia (Illumina Ovine SNP50v1), and 55 from Ireland (Illumina Ovine SNP50v1). Data from an additional 6 UK Suffolk (Illumina Ovine SNP50v3) were downloaded as provided by Kerr et al. [55], who described the samples as being from one region but they observed genotypic differentiation between the samples. After merging these datasets with the U.S. dataset and applying the quality controls described for the U.S. “reduced SNP” analyses, 12,242 SNP remained for analyses.

Analyses of international samples included genomic-based N_e_ estimates for each country using the LD method of NeEstimator [39]. The H_E_, Nei’s non-biased heterozygosity (Hnb), H_O_, and Wright’s F_IS_ for each country were computed using Genetix [56]. A PCA was performed in PLINK by computing a distance matrix and extracting the eigenvectors. Combinations of PC 1, 2, and 3 were visualized by country using the R software. The percentage of variation explained by each PC was estimated. Finally, model-based population structure was determined using ADMIXTURE, which was run for K = 2 to 15. After K = 15, the cross-validation error was still decreasing. Because 5 countries were included in the dataset, K = 2 to 5 were selected for visualization. The CLUMPP program was used to align and merge the replicates of the coancestry coefficient matrix, **Q**, and the STRUCTURE PLOT package was used to visualize the bar plots.

## Results

### Pedigree analyses

**Fig. 1:**
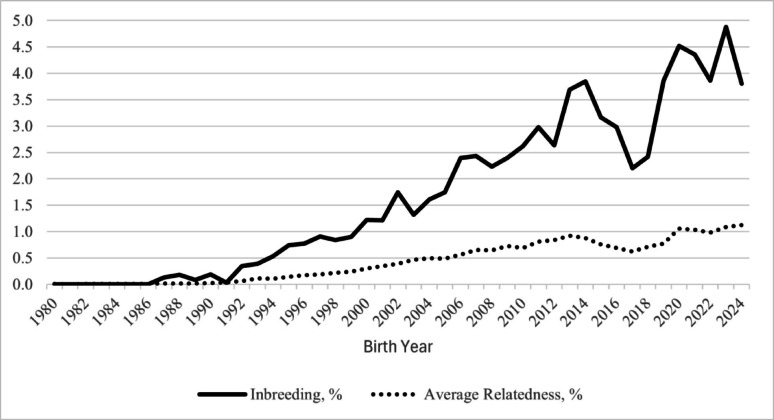
Inbreeding and average relatedness trends from 1980 to 2024 for U.S. Suffolk sheep

The summary statistics describing the full population and each subgroup are shown in Table 1. The F values ranged from 1.79% for the full pedigree to 5.41% for SG 2. The AR was low across the full population and subgroups, ranging from 0.46 to 1.25%. The F and AR trends since 1980 are plotted in Fig. 1 and show an increase in both F and AR over time.

**Table 1:** Summary statistics for the full population and subgroups (SG) 1, 2, 3, and 4 for U.S. Suffolk

Parameter	Full population	SG 1^a^	SG 2^b^	SG 3^c^	SG 4^d^
N	75,161	5,159	4,065	1,878	216
Flocks	128	28	25	16	27
F^e^, %	1.79	4.34	5.41	4.85	3.62
AR^f^, %	0.46	1.03	1.18	1.25	1.06
Mean maximum generations (maximum)	6.4 (24.0)	18.0 (24.0)	18.9 (24.0)	15.8 (23.0)	14.1 (21.0)
Mean complete generations (maximum)	1.6 (7.0)	2.7 (7.0)	3.2 (7.0)	2.8 (7.0)	2.4 (6.0)
Equivalent complete generations (maximum)	3.0 (12.7)	6.7 (12.4)	7.5 (12.4)	6.7 (12.4)	5.8 (11.6)
PCI^g^ at generation 5, %	22	64	67	71	55
*f*_*e*_^h^	504	216	177	158	186
*f*_*a*_^i^	300	74	58	49	67
*f*_*e*_ */ f*_*a*_	1.7	2.9	3.1	3.2	2.8
GCI^j^	5.2	9.5	10.4	7.8	7.5
Number of ancestors explaining 50% of genetic variation	178	36	27	24	35

^a^SG 1 = Subgroup 1, which included animals born from 2021 to 2023; ^b^SG 2 = Subgroup 2, which included SG 1 with a minimum 4-generation pedigree; ^c^SG 3 = Subgroup 3, which included genotyped animals; ^d^SG 4 = Subgroup 4, which included sires of SG 1; ^e^F = average individual coefficient of inbreeding; ^f^AR = average relatedness; ^g^PCI = pedigree completeness index; ^h^*f*_*e*_ = effective number of founders; ^i^*f*_*a*_ = effective number of ancestors; ^j^GCI = genetic conservation index

The overall generation interval was 2.78 ± 0.06 years based on the weighted mean of each of the four paths. The path for the sire-son was the shortest (2.29 ± 0.17 years), followed by sire-daughter (2.41 ± 0.05 years), mother-son (3.00 ± 0.14 years), and mother-daughter (3.20 ± 0.12 years). Based on the overall generation interval, the ΔF was 0.25% per generation and did not differ across generations (*P-value* = 0.49).

**Fig. 2:**
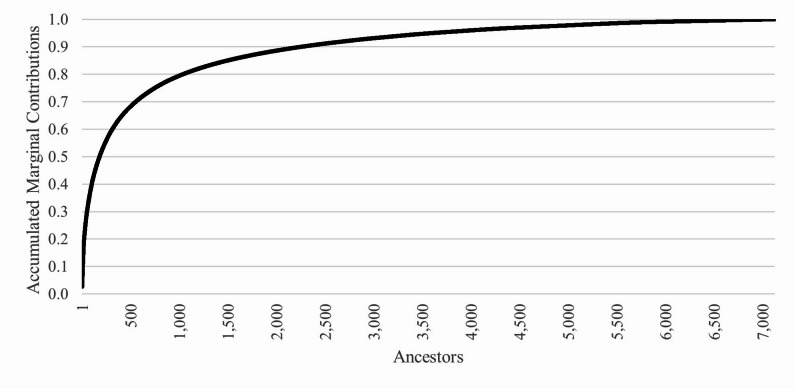
Accumulated marginal contributions by number of ancestors for U.S. Suffolk sheep

The accumulated marginal contributions of ancestors were plotted in Fig. 2. 50% of the genetic variation was explained by 178 ancestors. The top ten marginal contributors explained 13.1% of the genetic variation (Table 2). The top ten marginal contributors were all males and originated from 7 flocks. Only 7 rams contributed at least 1% of the genetic variation of the population; none of those 7 rams were currently active, with the most recent one born in 2005.

**Table 2:** Top 10 marginal contributors to the U.S. Suffolk gene pool

Animal rank	Marginal contribution	Accumulated contribution	Progeny	Flock	Birth Year
1	0.027	0.027	107	3	2005
2	0.020	0.047	257	7	2000
3	0.013	0.060	205	3	1998
4	0.013	0.073	135	17	2000
5	0.011	0.084	64	2	1994
6	0.010	0.094	92	7	1995
7	0.010	0.104	188	7	1995
8	0.009	0.113	55	5	2003
9	0.009	0.122	149	1	2001
10	0.009	0.131	118	18	1987

The seven pedigree-based N_e_ estimates ranged from 28 (increase in F by complete generation) to 194 (individual increase in coancestry) (Table 3). The median estimate was 80 (regression on birth year). For the 128 flocks in the pedigree, both the Wright’s F_ST_ and Nei’s distance were 0.026.

**Table 3:** Pedigree-based estimates of effective population size (N_e_) in the U.S. Suffolk population

Method	*N*_e_ estimate
Increase in F^a^ by maximum generation	182
Increase in F by complete generation	28
Increase in F by equivalent generation	56
Individual increase in F	85
Regression on birth year	80
Log regression of (1 – F) on generation number	44
Individual increase in coancestry	194

^a^F = average individual coefficient of inbreeding

### Genomic analyses

**Fig. 3:**
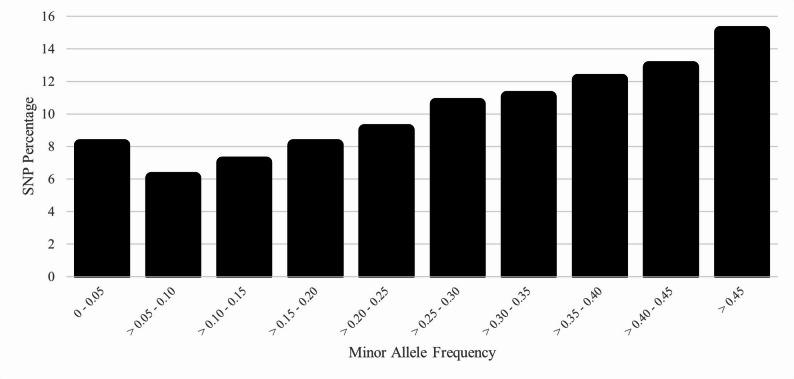
Distribution of single nucleotide polymorphism (SNP) frequency by minor allele frequency (MAF) category for the U.S. Suffolk population

**Fig. 4:**
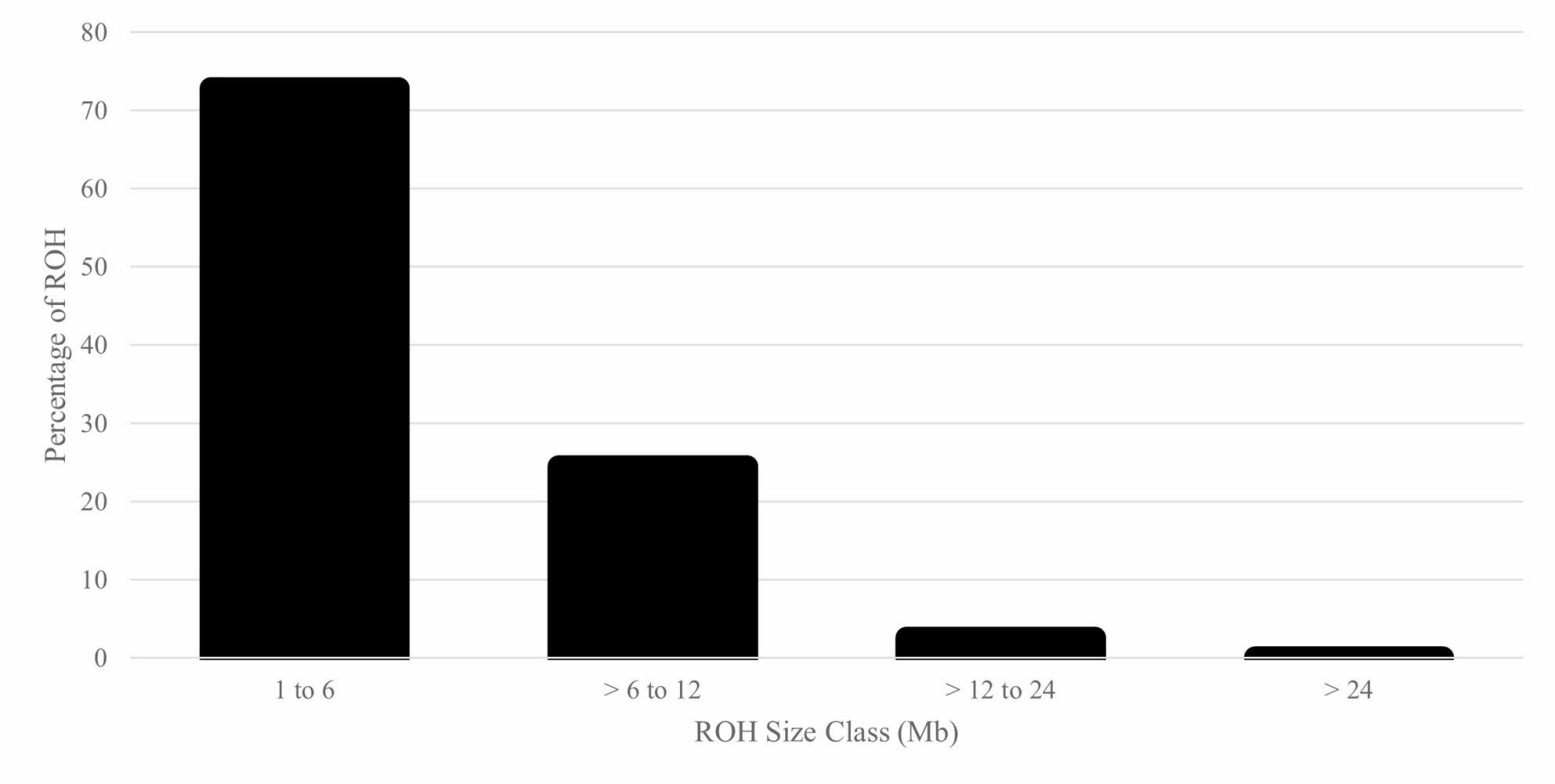
Percentage of runs of homozygosity (ROH) assigned to each size class for U.S. Suffolk sheep

**Fig. 5:**
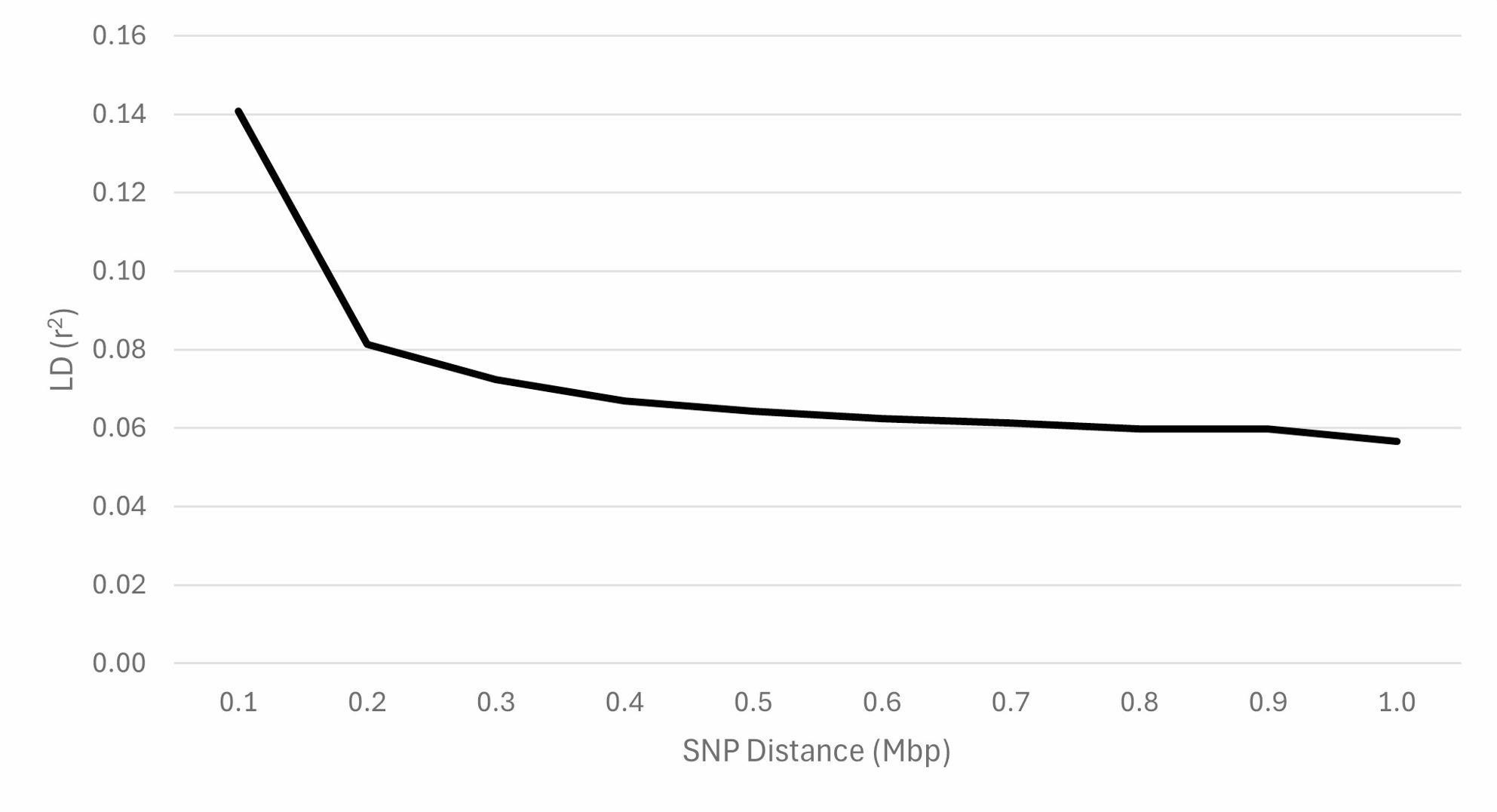
Linkage disequilibrium (LD) decay over increasing single nucleotide polymorphism (SNP) distance for U.S. Suffolk sheep

The genomic N_e_ estimates based on the reduced SNP dataset were 75 (jackknife confidence interval from 72 to 78) based on the NeEstimator software and 85 based on the GONE software. The distribution of MAF using the full SNP dataset demonstrated that 8% of the SNP were fixed or rare (MAF: 0 to 0.05) and 51% were highly polymorphic (MAF > 0.30) (Fig. 3). Using the reduced SNP dataset, the H_E_ was 0.379 and the H_O_ was 0.367. The mean F_IND_ was 3.3%. Based on the full SNP dataset, the mean F_ROH_ was 4.6% (SD = 4.2%) and ranged from 0.1 to 32.6%. Within each ROH class, the inbreeding was 3.0%, 1.3%, 0.2%, and 0.0% for 1 to 6, > 6 to 12, > 12 to 24, and > 24 Mb, respectively. The number of ROH per animal ranged from 0 to 81, with a mean of 14.8. The majority of ROH were short, with 73% in the 1 to 6 Mb class (Fig. 4). The F_GRM_ ranged from − 16.8 to 47.0% with an average of 3.3% (SD = 9.7%). The F_HBD_ ranged from 0 to 13.4% with a mean of 3.4% (SD = 1.8%). Based on the unpruned “LD dataset”, the overall LD among SNP pairs was low and generally declined with increasing distance between SNP pairs (Fig. 5). The average distance between adjacent SNP was 0.06 Mb, with an average LD (r^2^) of 0.15.

**Fig. 6:**
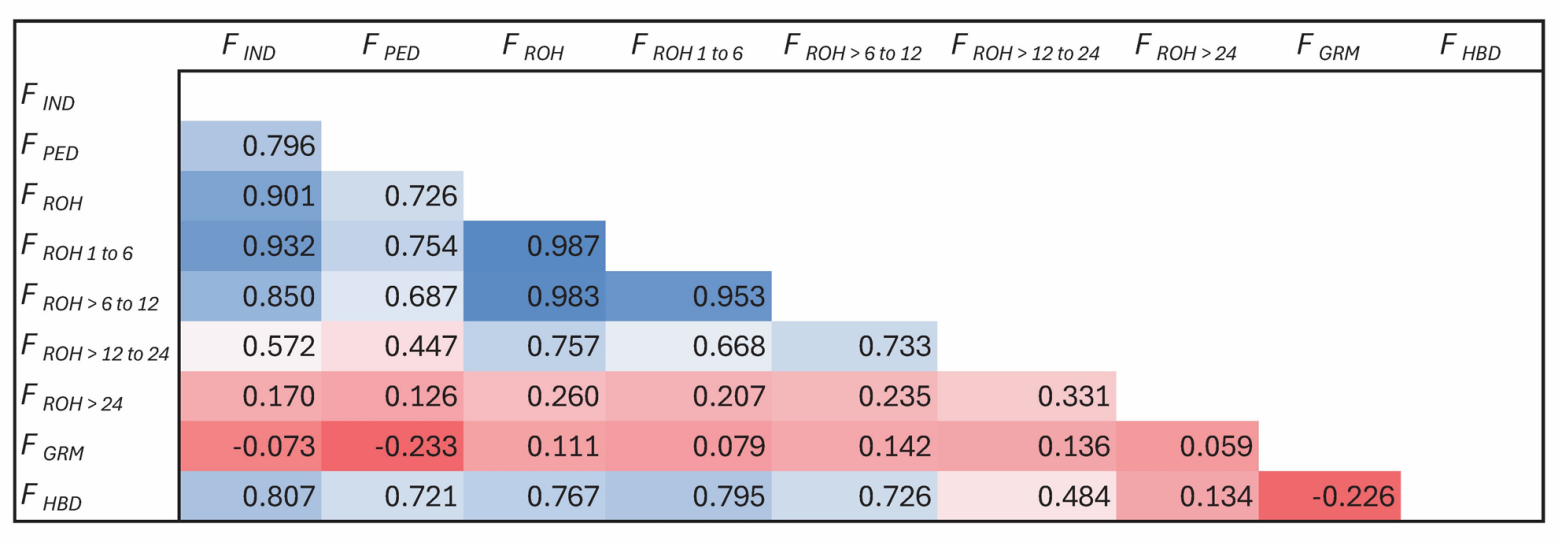
Pearson correlation coefficients between the inbreeding coefficients of genotyped U.S. Suffolk sheep based on heterozygosity F_IND_, pedigree (F_PED_), runs of homozygosity (ROH) for all class lengths (Mb) (F_ROH_), ROH classes 1 to 6 (F_ROH 1 to 6_), ROH classes > 6 to 12 (F_ROH > 6 to 12_), ROH classes > 12 to 24 (F_ROH > 12 to 24_), ROH classes > 24 (F_ROH > 24_), the genomic relationship matrix (F_GRM_), and homozygous-by-descent (F_HBD_)

Inbreeding was measured using five methods for the genotyped animals. Pedigree-based F was 4.8%, F_IND_ was 3.3%, F_ROH_ was 4.6%, F_GRM_ was 3.3%, and F_HBD_ was 3.4%. When comparing pedigree-based F to genomic-based F, the Pearson correlation coefficients ranged from a low for F_GRM_ to high for F_IND_ (Fig. 6). When comparing genomic-based measures, the Pearson correlation coefficients ranged from low between F_GRM_ and F_HBD_ to high between F_ROH_ and F_ROH 1 to 6_.

**Fig. 7:**
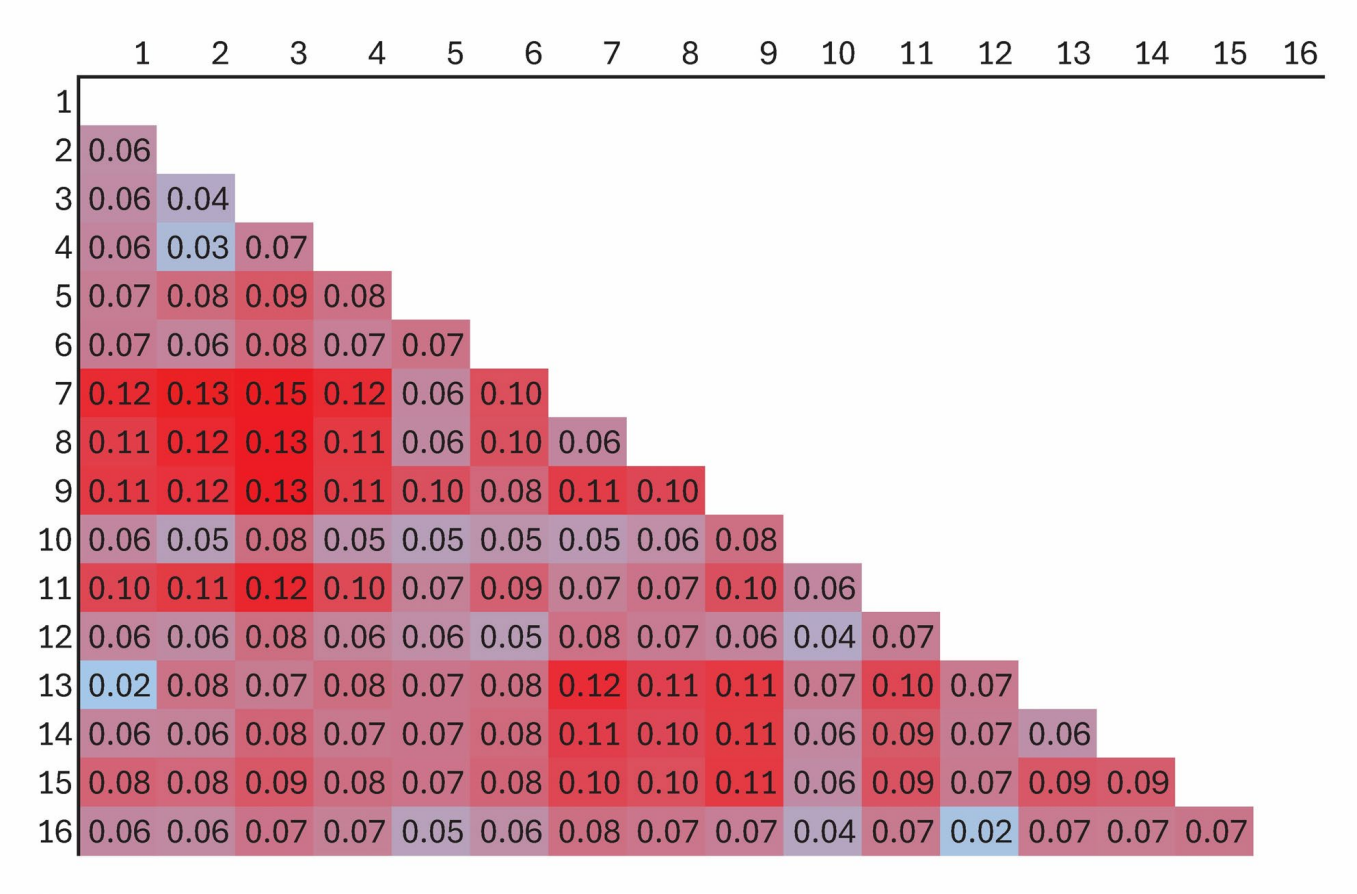
Heat map of fixation index (F_ST_) for Suffolk flocks

**Fig. 8:**
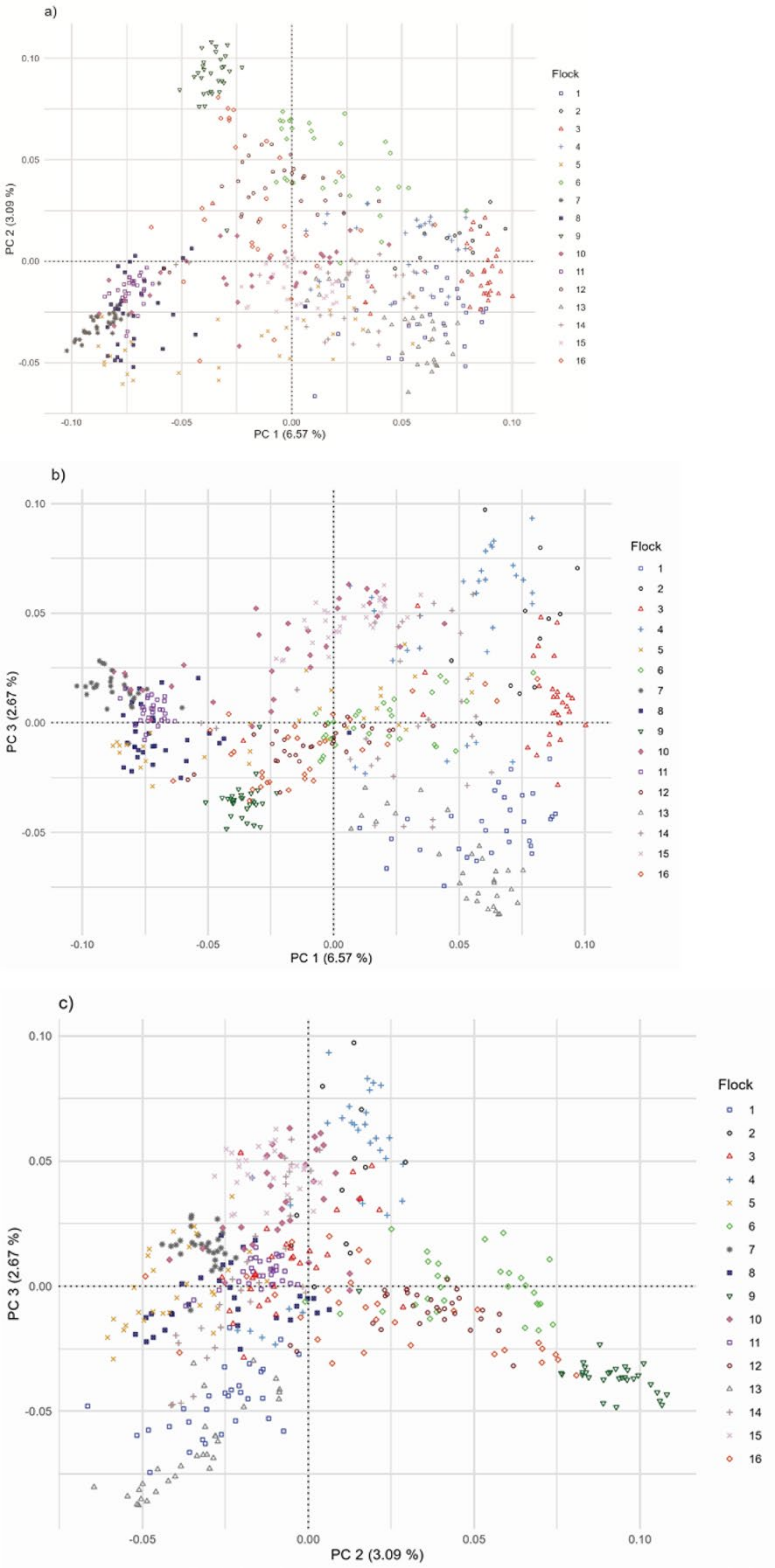
Plot of principal component 1 and 2 (**a**), 1 and 3 (**b**), and 2 and 3 (**c**) for U.S. Suffolk genotyped from 16 flocks (replicate 1)

**Fig. 9:**
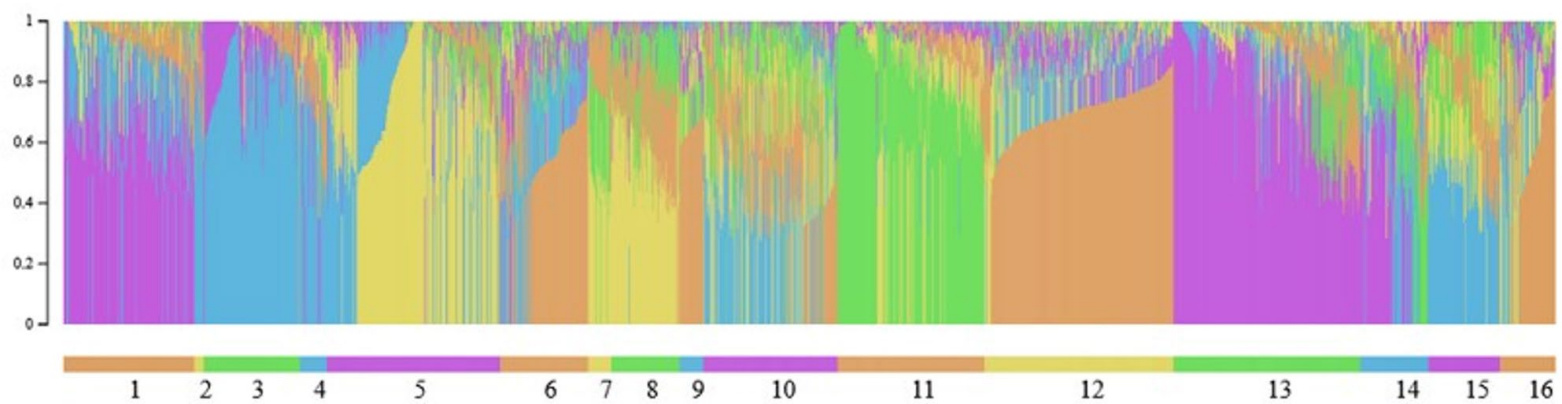
Model-based population structure for K = 5 for genotyped Suffolk, sorted by flock

The mean F_ST_ between the 16 flocks was 0.08 (Fig. 7). Flocks 7, 8, 9, and 11 had the highest F_ST_ in comparison with the other flocks. These four flocks show differentiation in the PCA plots, with flock 9 separating from the others (Fig. 8; see Additional file 1 Fig. S1; Additional file 2 Fig. S2; Additional file 3 Fig. S3; Additional file 4 Fig. S4). The variation explained by each PC was consistently low across replicates and ranged from 6.43 to 6.85% for PC 1, 3.09 to 3.23% for PC 2, and 2.64 to 2.82% for PC 3. The model-based population structure did not show the same level of differentiation for flocks 7, 8, 9, and 11 as observed with the F_ST_ values and the PCA (Fig. 9, Additional file 5 Fig. S5; Additional file 6 Fig. S6). There was considerable admixture observed across all flocks.

### International comparison

**Fig. 10:**
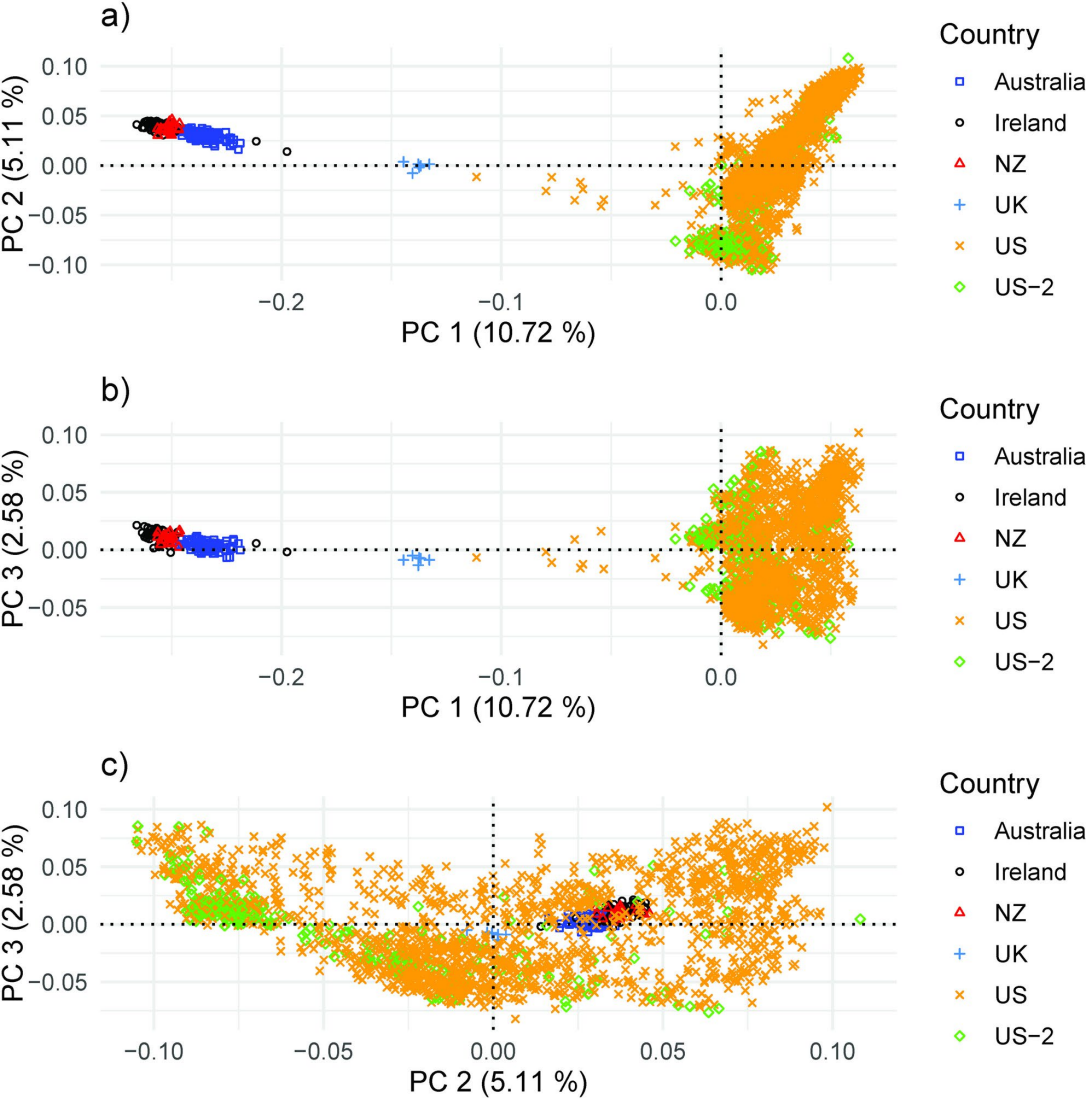
Plot of principal component 1 and 2 (**a**), 1 and 3 (**b**), and 2 and 3 (**c**) for Australian, Irish, New Zealand, United Kingdom, and U.S. Suffolk

The LD-based N_e_, H_E_, Hnb, H_O_, and F_IS_ for the Suffolk population from each country represented are summarized in Table 4. For the U.S. data, the N_e_ using the NeEstimator software based on the reduced dataset (34,243 markers) was 75.0 and the N_e_ based on the international dataset (12,242 markers) was 75.1, suggesting that the reduced number of markers for the international comparison should not influence the results. However, the overall results based on the international dataset should be used with caution. The sample available in some countries was small, and potentially did not reflect the full extent of genetic diversity in its Suffolk population. Australia had the highest H_E_ and H_O_. Australia and the United Kingdom had a negative F_IS_. Ireland had the highest F_IS_. The PC were plotted by country for combinations of PC 1, PC 2, and PC 3 (Fig. 10). Each PC explained 10.7%, 5.1%, and 2.6% of the variation, respectively. For PC 1 versus PC 2 and PC 1 versus PC 3, Australia, Ireland, and New Zealand were grouped together with the United Kingdom intermediate between those three countries and the U.S. In PC 2 versus PC 3, the U.S. is dispersed across the plot with the United Kingdom separated from the clustering of Australia, Ireland, and New Zealand.

**Fig. 11:**
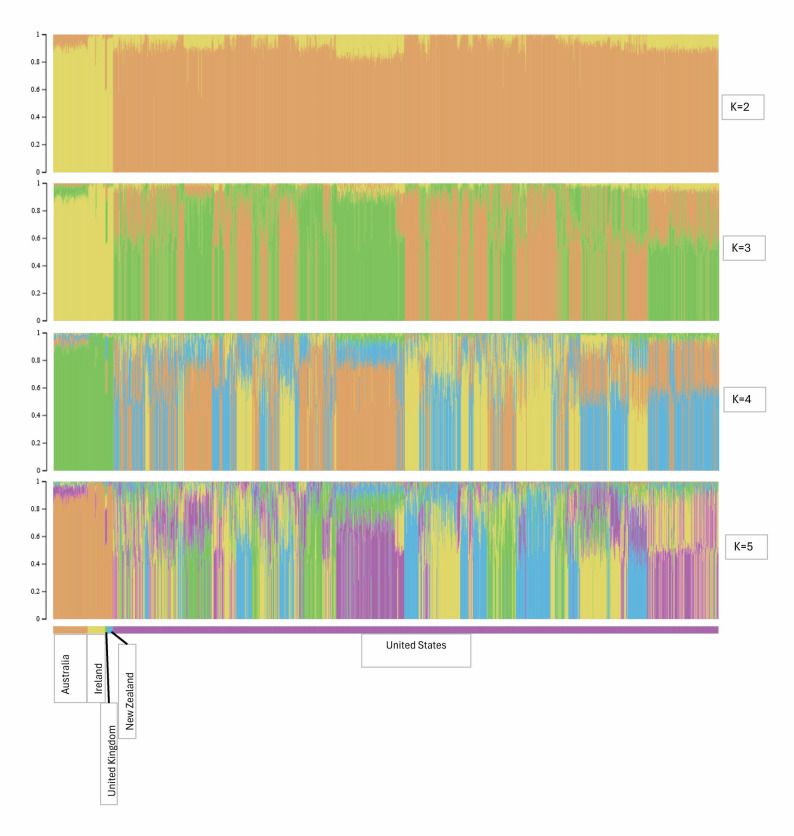
Model-based population structure for K = 2 to 5 for genotyped Australian, Irish, New Zealand, United Kingdom, and U.S. Suffolk, sorted by country

The model-based population structure mirrors the PCA results (Fig. 11). For all K (2 to 5), Australia, Ireland, and New Zealand display a similar proportional assignment to each subpopulation, the United Kingdom displays an intermediate level of admixture, while the U.S. shows considerable and varying admixture. A plot of the cross-validation errors for the subpopulations is provided in Additional file 7 Fig. S7.

**Table 4:** The effective population size (N_e_), expected heterozygosity (H_E_), nei’s non-biased heterozygosity (Hnb), observed heterozygosity (H_O_), and wright’s F_IS_ for Suffolk sheep in five countries

Country	*N*	*N*_e_	H_E_	Hnb	H_O_	F_IS_
Australia	109	101.9	0.427	0.429	0.429	-0.001
Ireland	55	22.0	0.380	0.383	0.364	0.050
New Zealand	19	667.4	0.379	0.389	0.388	0.002
United Kingdom	6	34.9	0.360	0.393	0.412	-0.053
U.S.	1,878	75.1	0.392	0.392	0.380	0.032

## Discussion

The Suffolk breed is an important terminal sire breed throughout the U.S. Suffolk rams are used extensively to generate terminal crossbred offspring on large commercial range sheep operations in the Western U.S. in addition to their use on smaller flocks throughout the rest of the country. Previous studies have characterized genetic differences within the U.S. Suffolk either by region [38] or by differing breed objectives [9, 57]. Of the Suffolk sheep breeders throughout the U.S., only a subset (less than 50%) is actively participating in NSIP. However, these are the breeders that will likely be engaged in GS moving forward, and thus the focus of a baseline measure of genetic diversity and population structure.

### Genetic diversity

Wilson et al. [9] previously analyzed genetic diversity in the U.S. Suffolk breed, which included the NSIP pedigree and both NSIP and non-NSIP Suffolk sheep (*n* = 304) genotyped with the OvineHD BeadChip (Illumina Inc., San Diego, CA, USA). In the present study, an additional 10,851 animals (157 sires and 1,228 dams) were added to the pedigree, including historic data from a large flock that contributed the most genotyped animals. Of the 304 genotyped animals in the previous study, 244 were included in the current one, contributing 13% of the total animals. Although 28,829 markers are present on both the OvineHD and GGP Ovine 50 K arrays, only 7.4% and 62.2% of the markers from the OvineHD array and GGP Ovine 50 K array, respectively, were included in the analyses after data filtering and LD pruning. This left 2,154 markers that overlapped between the previous and present study. The OvineHD array provided a higher genotypic resolution throughout the genome compared to the GGP Ovine 50 K array, which is expected to have an impact on the results.

When comparing the smaller dataset to the present study, pedigree-based inbreeding, pedigree-based N_e_, and genomic N_e_ were similar. The *f*_*e*_ and *f*_*a*_ were higher in the present study and likely reflect greater pedigree depth and breadth. When comparing flocks, two of the three flocks that were differentiated in the earlier study (F_ST_, PCA, model-based population structure) were also differentiated in this study (flocks 9 and 11). A PCA comparison of the overlapping 13% of animals by flock for the previous study and the current study (Additional file 8 Fig. S8), showed a similar pattern of differentiation of flocks regardless of the genomic markers used. Differentiation among flocks is likely due to differing breeding objectives rather than physical distance between flocks. Some breeders have selected for a body type that excels at show competitions while others have prioritized producing terminal-sired lambs for market. Although both body type extremes are present in the U.S. Suffolk breed, some breeders produce rams that are intermediate and can be used in either the show ring or for producing market lambs.

As part of the international comparisons, the Australian and Irish datasets were used in both the current and previous study [9] of Suffolk genetic diversity. The LD based estimates of N_e_ were similar between the studies. Based on PCA, the U.S. had greater dispersion than the other countries in both studies, which may be at least partially due to the greater number of U.S. animal included in the study. A PCA comparing the overlapping 13% of U.S. Suffolk from both studies to Australian and Irish Suffolk shows clear separation between the three countries (Additional file 9 Fig. S9). For the model-based population structure, Australia and Ireland were differentiated in the earlier study but were grouped together in the current one, which is likely due to the much larger U.S. dataset and the inclusion of data from additional countries. Further, the genotypic resolution was different among the two arrays, which is expected to influence the results. The animals that were also included in the previous study were separated from those animals to form two U.S. populations of NSIP Suffolk (Additional file 10 Fig. S10). The H_E_, Hnb, H_O_, and F_IS_ were then re-computed for the two U.S. subsets and the international countries. For the U.S. Suffolk in the previous study, H_E_, Hnb, H_O_, and F_IS_ were 0.396, 0.397, 0.386, and 0.027, respectively, and 0.390, 0.390, 0.379, and 0.030 for the additional animals in the present study, demonstrating consistent results across studies.

The generation interval of 2.8 years was slightly lower than reported previously in U.S. (2.9 years [9]) and Canadian (3.3 years [58]) Suffolk. Of the 4 paths used to compute the generation interval, the paths (a parent to a son or to a daughter) were shorter for sires (2.3 and 2.4 years, respectively) than dams (3.0 and 3.2 years, respectively). Sire paths are expected to be shorter as mature rams are replaced by younger rams with improved genetics. Conversely, dam paths are expected to be longer as ewes are retained to maximize their productive life. The generation interval in Suffolk is shorter than that reported for other breeds [8, 59, 60], which may reflect a more rapid replacement by improved animals, the likely lower longevity of the breed’s animals [61–63], or both.

The pedigree-based F was 4.3% for SG 1 and 5.4% for SG 2. Using the same criteria to define the subgroups in composite sheep breeds, the U.S. Polypay and U.S. Katahdin had a lower pedigree-based F for both SG 1 and SG 2 [6, 8]. The pedigree-based F for Canadian Suffolks born in 2010 was also lower [58]. Other genomic reports of F in U.S. Suffolk were 3.5% and 13–14% for F based on heterozygosity [9, 38]. For other U.S. sheep breeds, F_IND_ was lower for Polypay and F_ROH_ was higher for both Polypay and Katahdin [7, 8].

Pearson correlations between pedigree-based F with F_IND_, F_ROH_, F_ROH_ classes of 1 to 6 and > 6 to 12, and F_HBD_ were high. The shorter ROH classes are associated with past inbreeding so the high correlation with pedigree-based F is unexpected. Although short ROH segments are typically attributed to ancient inbreeding, their strong correlation with pedigree-based F may reflect persistent background autozygosity as well as the inability of pedigrees to fully capture deeper ancestral contributions. Interestingly, Ferenčaković et al. [64] observed that even in breeds with relatively complete pedigrees, shorter ROH classes (e.g., > 1 Mb and > 2 Mb) maintained moderate-to-high correlations with pedigree-based F, suggesting that pedigree-based inbreeding coefficients can partially track more distant autozygosity when deep relatedness exists within structured populations. The correlation between pedigree-based F and F_GRM_ was negative (− 0.23). With F_GRM_, the homozygous genotypes are weighted such that low frequency homozygous genotypes contribute more to the inbreeding measure than high frequency homozygous genotypes with the aim of identifying alleles that are identity-by-descent (IBD) rather than identity-by-state (IBS) [65]. According toVillanueva et al. [65], computation of the F_GRM_ is useful for genomic selection, which was the purpose for inclusion in this analysis, but may not be the best measure of inbreeding. The correlations between F_ROH_ and the F_ROH_ classes decreased with increasing class size, which may be explained by fewer ROH events being assigned to the larger class sizes rather than the data structure. The correlation between F_ROH_ and F_GRM_ was low; this is not unexpected since, as described above, the adopted GRM (based on VanRaden’s first method) is more dependent on the estimates of allele frequency in the population used for scaling, which could be biased depending on the genotyping array, sample size, and the genotyping sampling strategy [43, 66]. Since both ROH and HBD are measures of homozygous segments in the genome, the high correlation between F_ROH_ and F_HBD_ provides confirmation that the ROH parameters were adequate to capture the homozygosity present in the genome for each animal included in the analyses.

The rate of inbreeding to maintain a sustainable population is suggested to be less than 1% per generation [67] and the U.S. Suffolk was well below this rate (0.25%). Inbreeding was concentrated within the smaller ROH classes, which indicates past rather than current inbreeding. In comparison with Canadian Suffolk, U.S. Polypay, and U.S. Katahdin, the U.S. Suffolk had a higher *f*_*e*_, *f*_*a*_, and number of ancestors contributing to 50% of the gene pool [6, 8, 58]. When comparing the marginal contributors to the population, none of the sires were highly influential and they were all born in early generations. This suggests that there were no dominant sires or sire lines in the current population.

The true N_e_ of a population has multiple uses, including for breed conservation, development of a reference population for GS, and predictions of accuracies for GS. However, computing the true N_e_ is challenging because the concept is based on an idealized population, which does not exist in animal breeding programs. Consequently, multiple computation strategies can be used to provide a range of pedigree-based N_e_ values instead of a single value. To maintain the genetic fitness of a population, an acceptable N_e_ is a minimum of 50 [67, 68] to 100 [69]. Both the genomic- and pedigree-based estimates of N_e_ were lower for Suffolk than for U.S. Polypay and U.S. Katahdin using the same methods and SNP assay [6–8]. Two of the pedigree-based measures were below 50 and only two measures were above 100. The genomic-based N_e_ were 75 and 85, which are approaching a concerning level for preventing a decline in fitness [69, 70]. Although the majority of SNP were highly polymorphic (MAF > 0.30), the H_E_ and H_O_ of the U.S. Suffolk were lower than for U.S. Polypay and similar to U.S. Katahdin [7, 8] using the same SNP array. However, this may be expected since the Polypay and Katahdin are relatively new composite breeds. The H_E_ and H_O_ were also lower than other breeds using the GGP Ovine 50 K BeadChip, including Czech Sumava, Czech Wallachian, and Slovenian Valachian [71, 72].

The LD between the markers and quantitative trait loci (QTL) of interest play an important role if these markers are to be used in selection decisions [73–75]. The LD decay with increasing marker distance of the Suffolk population was like that reported for other breeds, including the U.S. Polypay and New Zealand Coopworth, Perendale, and Romney [8, 76]. The low LD observed in sheep breeds is in contrast to much higher values reported for dairy, beef, and pigs [77–79], making GS and genome-wide association studies with the genome coverage provided by a 50 K SNP array more challenging. This amplifies the need in species such as sheep with lower LD to carefully develop and update the reference population or genotype (or re-sequence) greater number of SNPs (higher density SNP arrays or re-sequencing methods). Nonetheless, improvements in accuracy of breeding value estimates have been achieved with a 50 K SNP assay [80–82].

There was evidence of population structure, with differentiation between flocks based on F_ST_ and PCA. Four flocks exhibited greater pairwise F_ST_ and more separation in the PCA plots. Flock 9 separated from the other flocks more consistently in the PCA. However, this differentiation was not present in the model-based population structure, where a high degree of admixture was found across all flocks. Population structure provides an opportunity to maintain outcross subpopulations within a breed while admixture provides genetic ties across flocks. While the U.S. Suffolk exhibits some population structure, the genetic diversity within the breed is more defined by admixture than by differentiation.

### International comparison

While GS is expected to be implemented through NSIP for U.S. Suffolk sheep, independent from other populations, it is important to quantify the genetic diversity available for this internationally important breed. Despite the high levels of heterozygosity present in the Australian, Irish, and New Zealand populations, they tended to cluster together both in the PCA plots and the model-based population structure plots. When comparing PC 2 to PC 3, the U.S. Suffolk was dispersed across the entire plot. This dispersion was observed when comparing goat breeds from Canada to Australia and to Europe [42, 83]. Close clustering within a country combined with separation from another country may indicate different breeding objectives in the U.S. compared to Australia and New Zealand. The Suffolk sheep from the United Kingdom may appear intermediate between the other countries because it was the origin of the breed, which then diverged due to differences in selection practices within those countries.

### Sample size

Genetic diversity studies have been performed with 10 or fewer animals representing a breed [84–88]. While sample size for building a reference population for genomic studies has been discussed extensively [89–92], there are no guidelines for which animals or how many animals to include in genetic diversity studies. In general, the previous study of U.S. Suffolk sheep including 304 genotyped animals seems to be as effective when evaluating pedigree-based F, pedigree-based N_e_, and genomic N_e_ as the present study including 1,878 animals. However, increasing sample size and, in particular, the number of sampled flocks will allow for greater evaluation of population structure within the breed.

## Conclusions

As a key outcome of this study, genetic diversity and population structure for the U.S. Suffolk have been quantified in the current NSIP population prior to implementation of GS. While the rate of inbreeding is acceptable, measures of N_e_ are lower than for other U.S. sheep breeds. Population structure exists within the breed, but more admixture was observed than differentiation. This admixture provides genetic ties for genetic evaluation but does not provide the safeguard of outcross populations with unique genetic diversity. Since the population of Suffolk sheep evaluated included only animals from NSIP-participating flocks, the opportunity exists to infuse new genetics from non-NSIP flocks. Also, while more challenging to achieve, Suffolk sheep from other countries are differentiated from the U.S. Suffolk and could provide a reservoir of new genetic diversity.

## Supplementary Information

Below is the link to the electronic supplementary material.

Supplementary Material 1.

Supplementary Material 2.

Supplementary Material 3.

Supplementary Material 4.

Supplementary Material 5.

Supplementary Material 6.

Supplementary Material 7.

Supplementary Material 8.

Supplementary Material 9.

Supplementary Material 10.

## Data Availability

The raw data cannot be made available as they are the property of the sheep producers participating in the NSIP, and this information is commercially sensitive. Requests to access these datasets should be directed to Dr. Carrie Wilson (e-mail: carrie.wilson@usda.gov).
