# Supplementary figures and images for: Genetic diversity and population structure of U.S. Suffolk sheep participating in the national sheep improvement program
Source: Genet Sel Evol. 2026 Jan 14;58:7. doi: 10.1186/s12711-025-01027-4 (PMC12809957; doi:10.1186/s12711-025-01027-4)

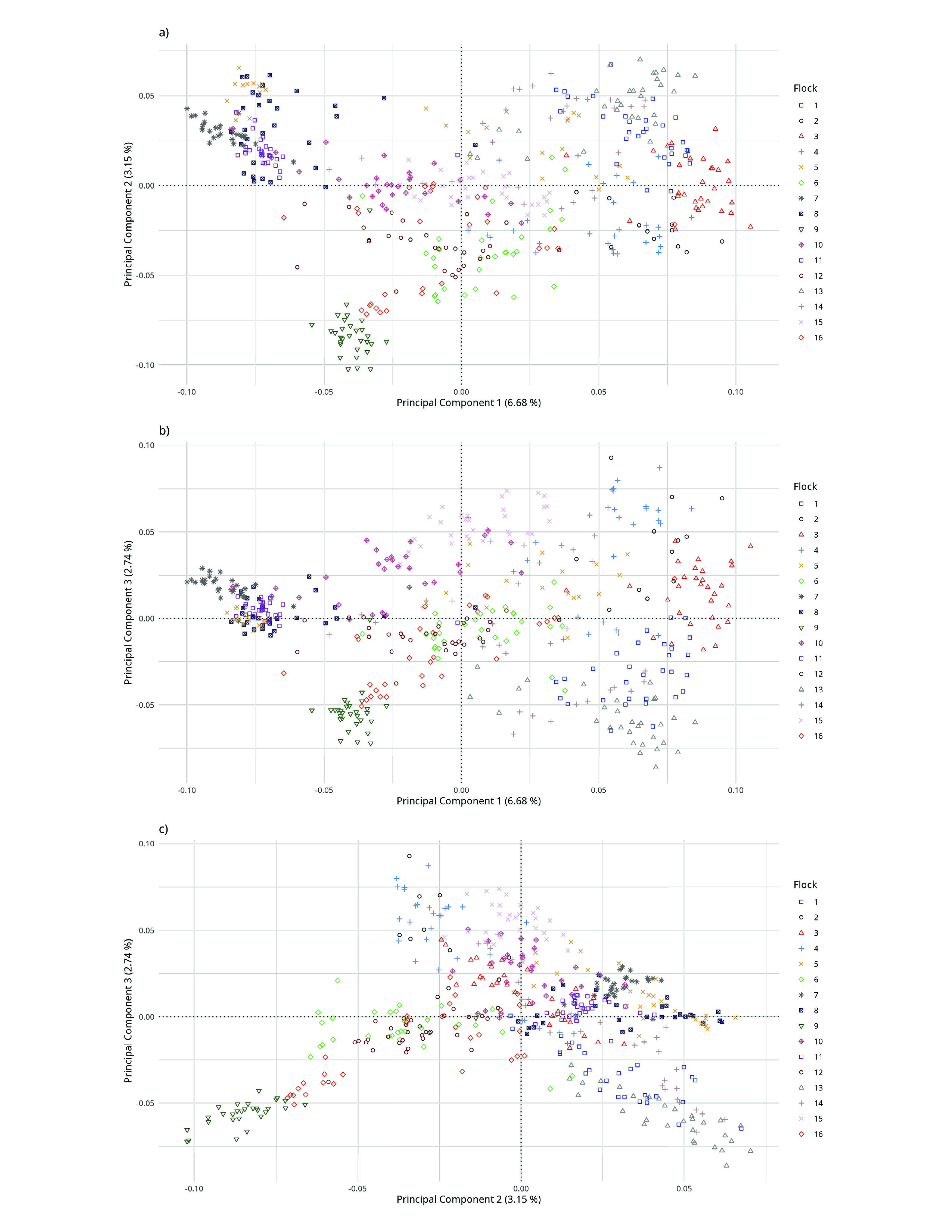

Supplement: Supplementary file 1 — Supplementary Material 1. [file 12711_2025_1027_MOESM1_ESM.jpg]

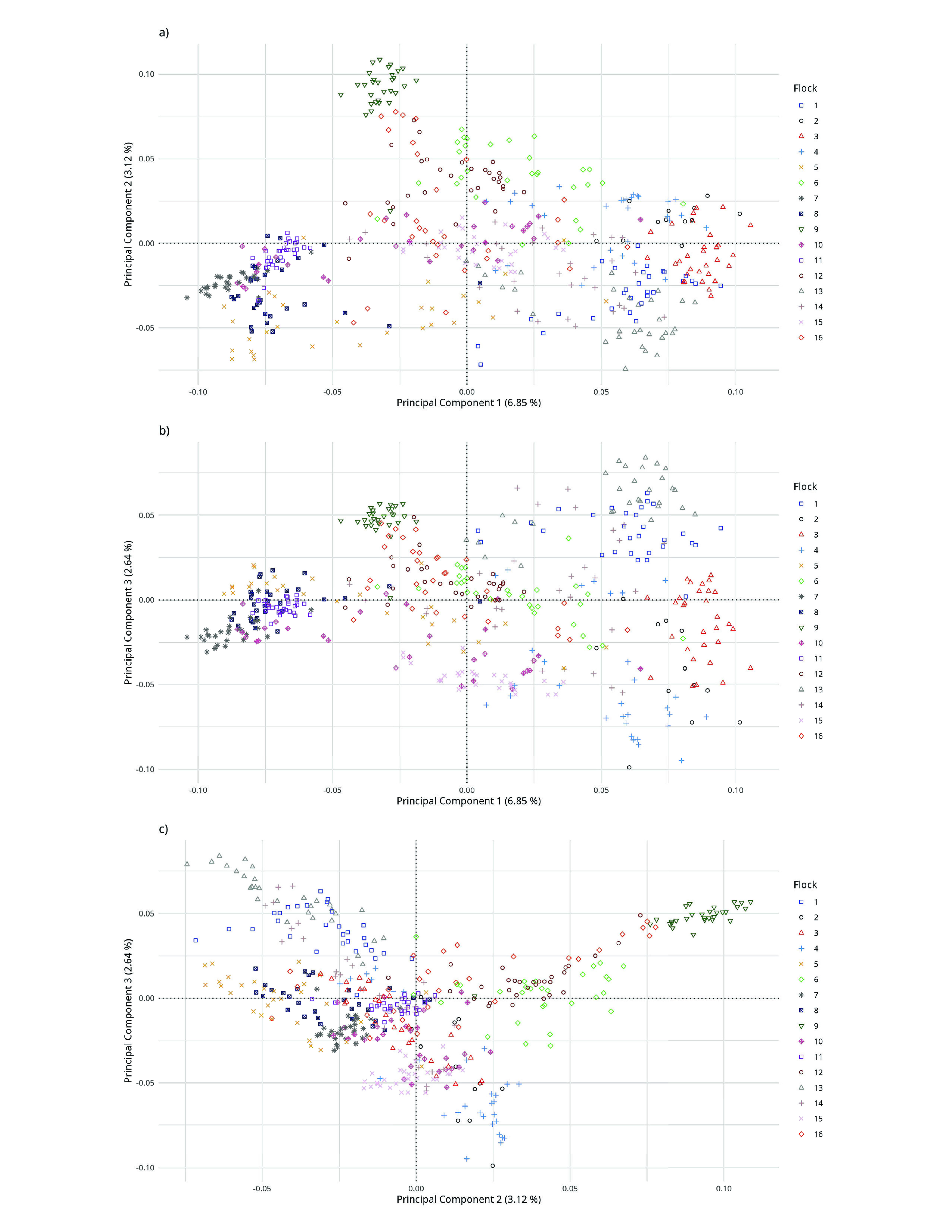

Supplement: Supplementary file 2 — Supplementary Material 2. [file 12711_2025_1027_MOESM2_ESM.jpg]

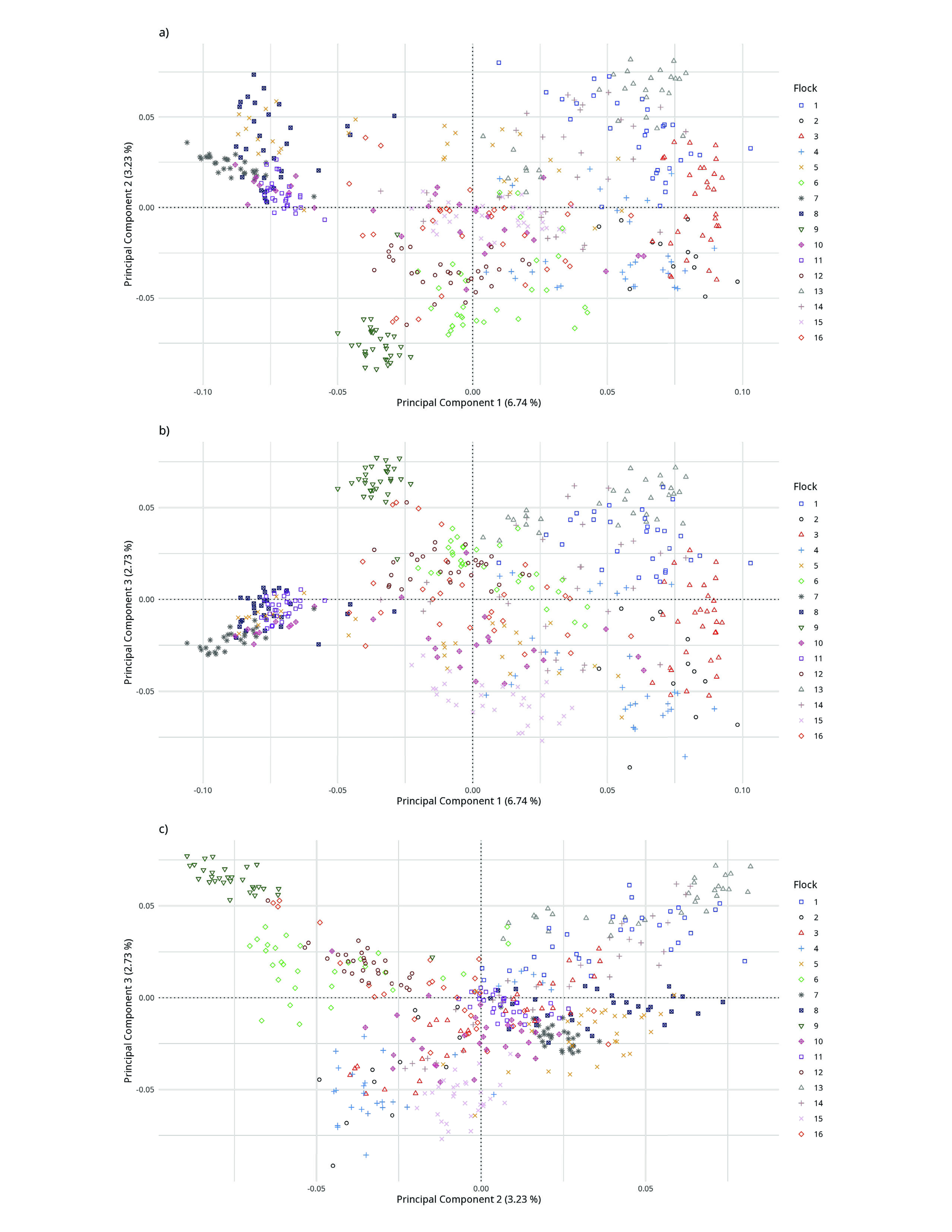

Supplement: Supplementary file 3 — Supplementary Material 3. [file 12711_2025_1027_MOESM3_ESM.jpg]

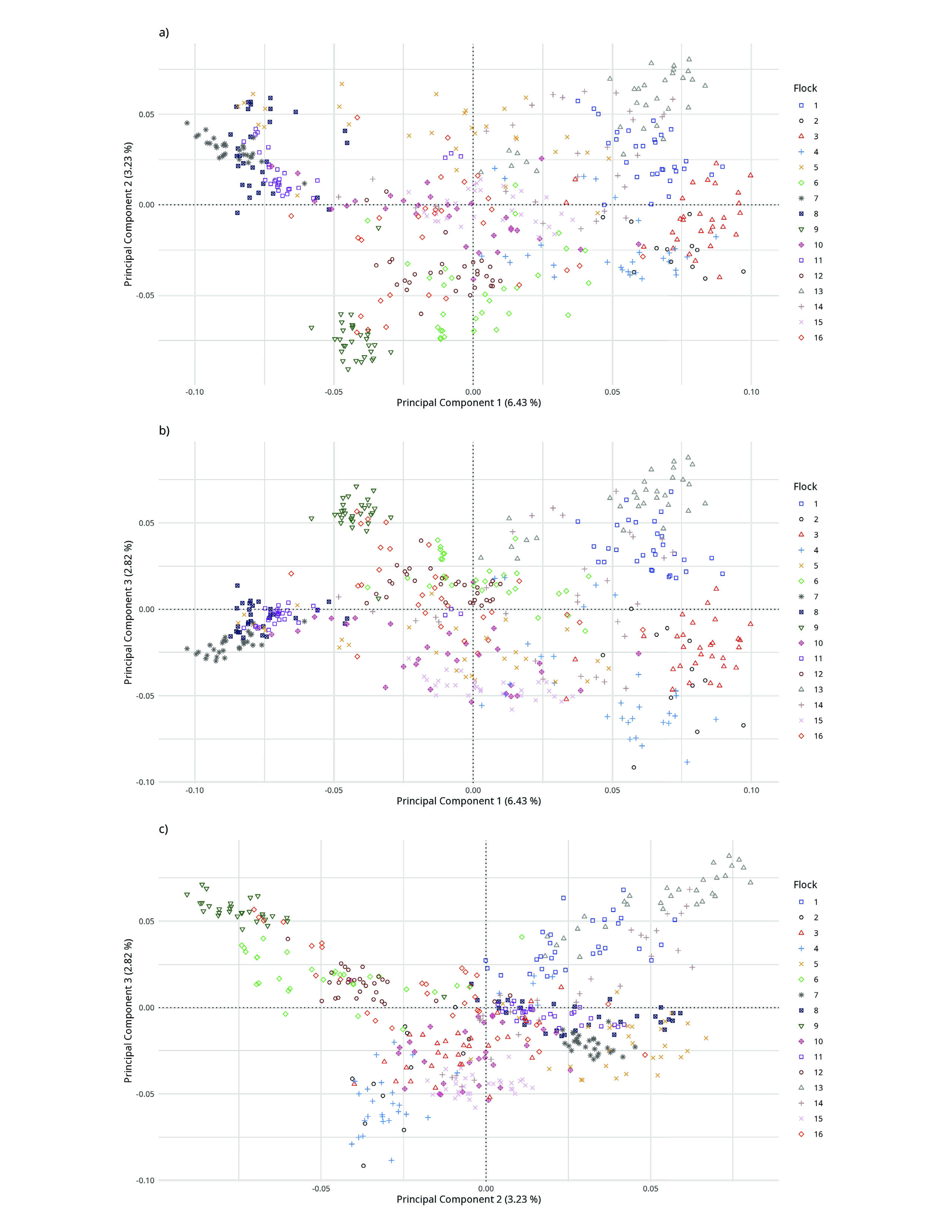

Supplement: Supplementary file 4 — Supplementary Material 4. [file 12711_2025_1027_MOESM4_ESM.jpg]

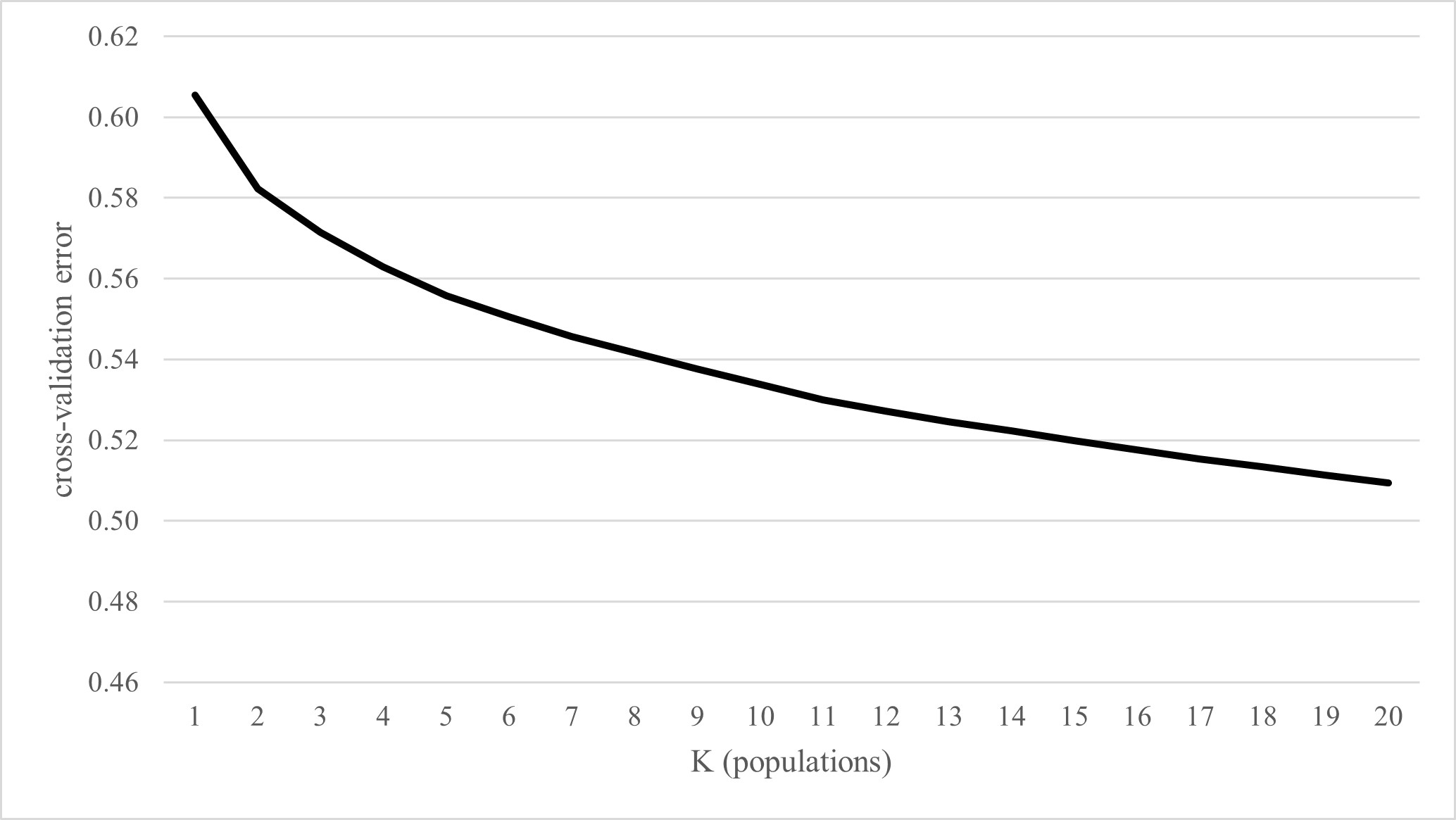

Supplement: Supplementary file 5 — Supplementary Material 5. [file 12711_2025_1027_MOESM5_ESM.jpg]

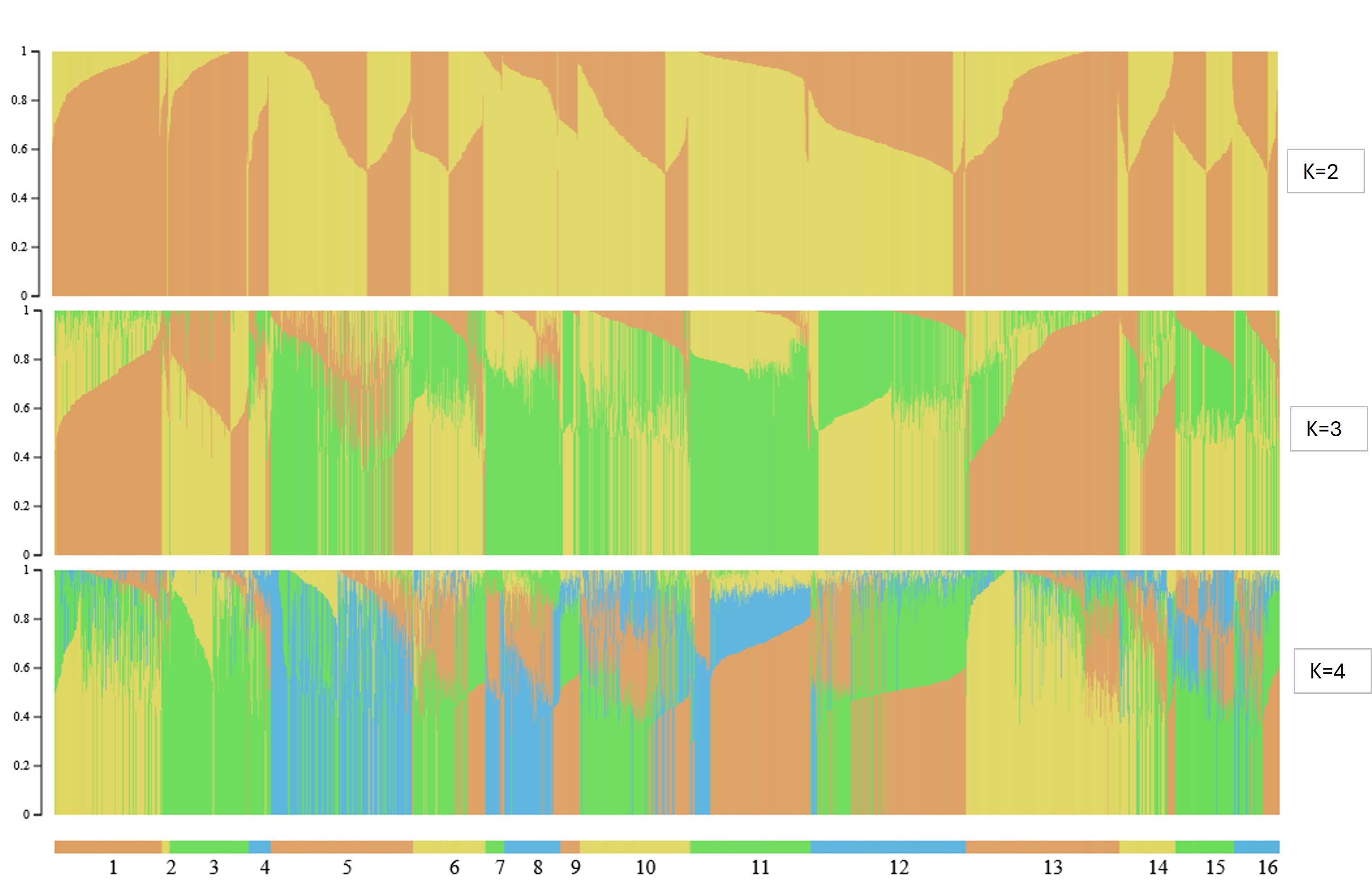

Supplement: Supplementary file 6 — Supplementary Material 6. [file 12711_2025_1027_MOESM6_ESM.jpg]

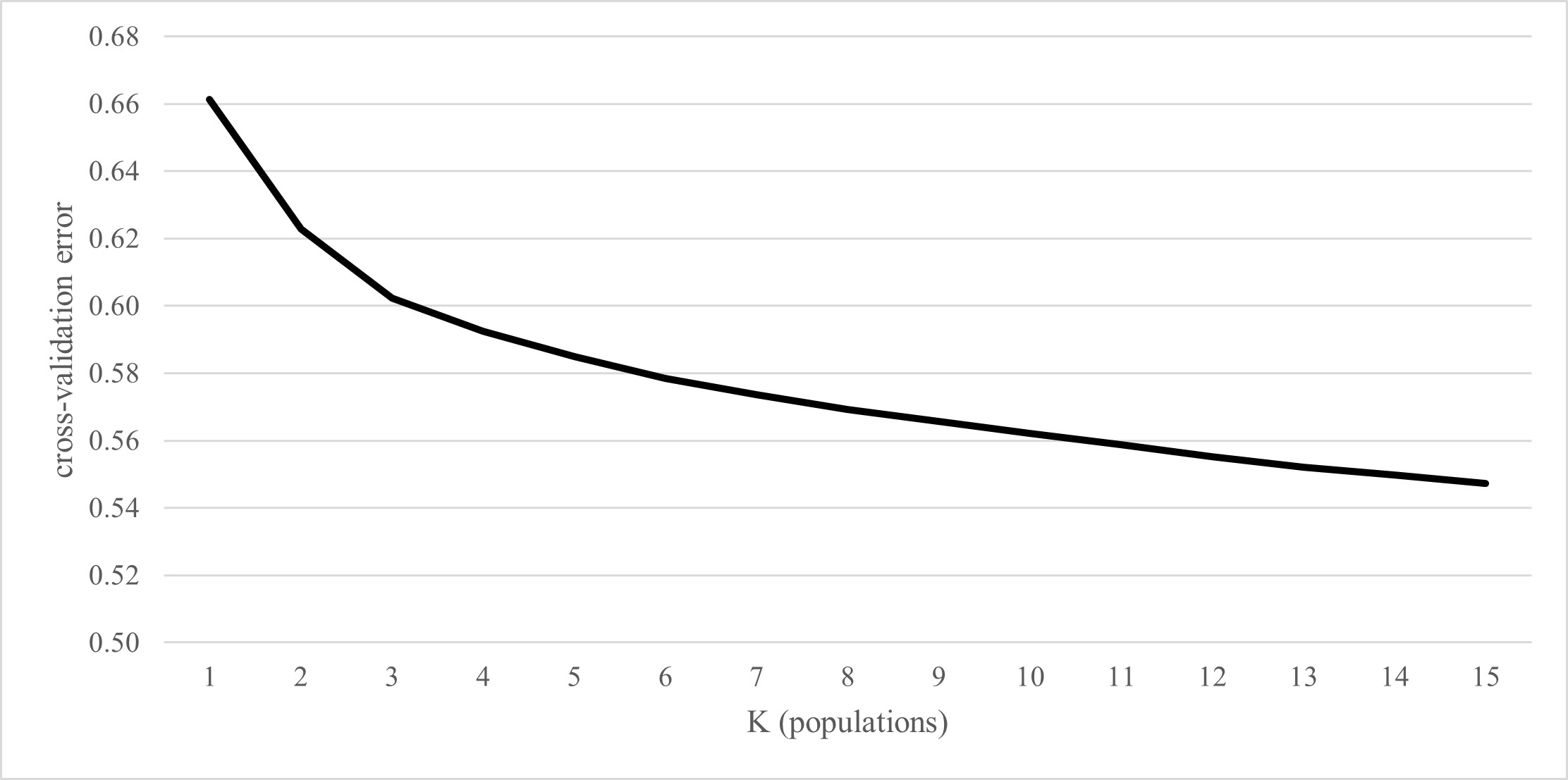

Supplement: Supplementary file 7 — Supplementary Material 7. [file 12711_2025_1027_MOESM7_ESM.jpg]

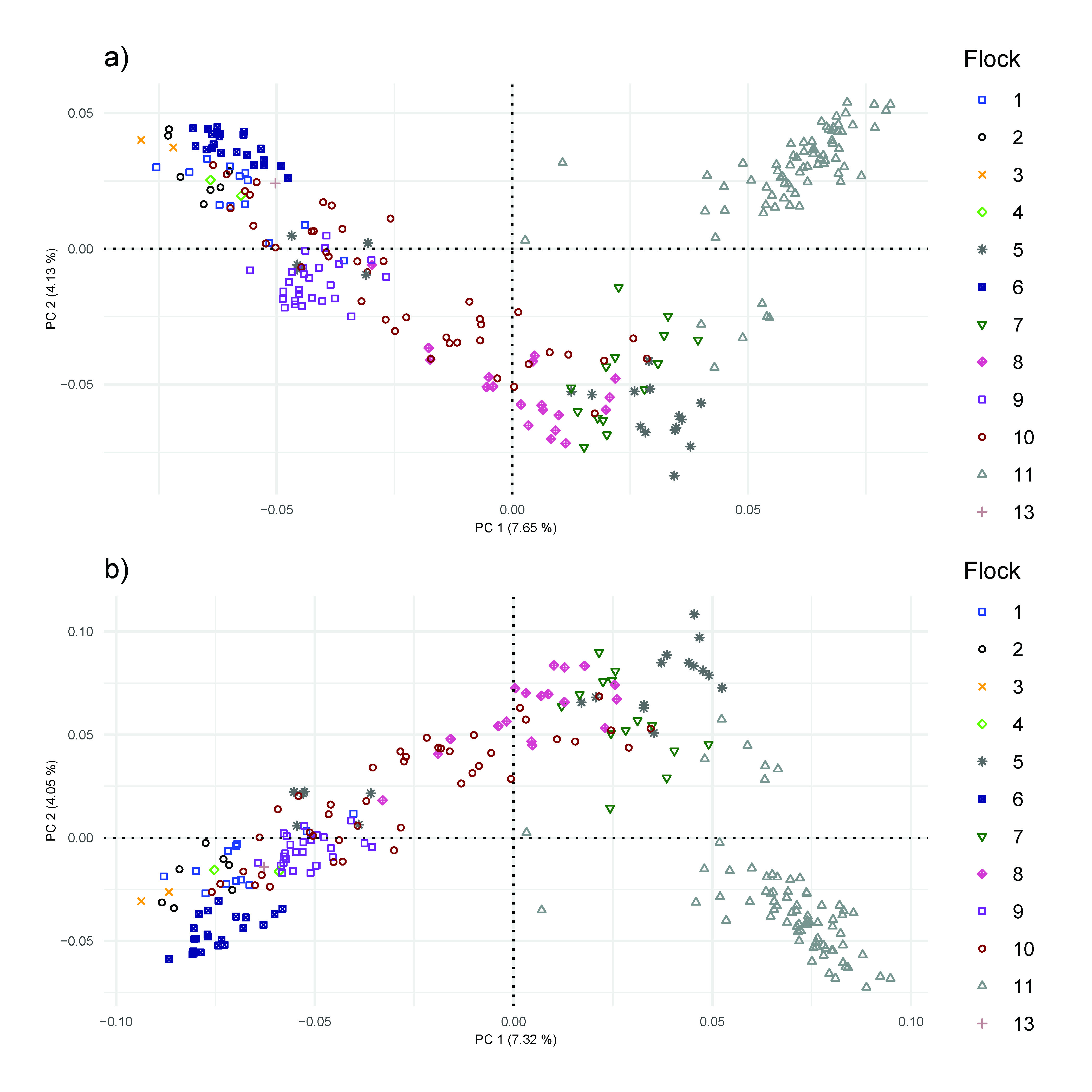

Supplement: Supplementary file 8 — Supplementary Material 8. [file 12711_2025_1027_MOESM8_ESM.jpg]

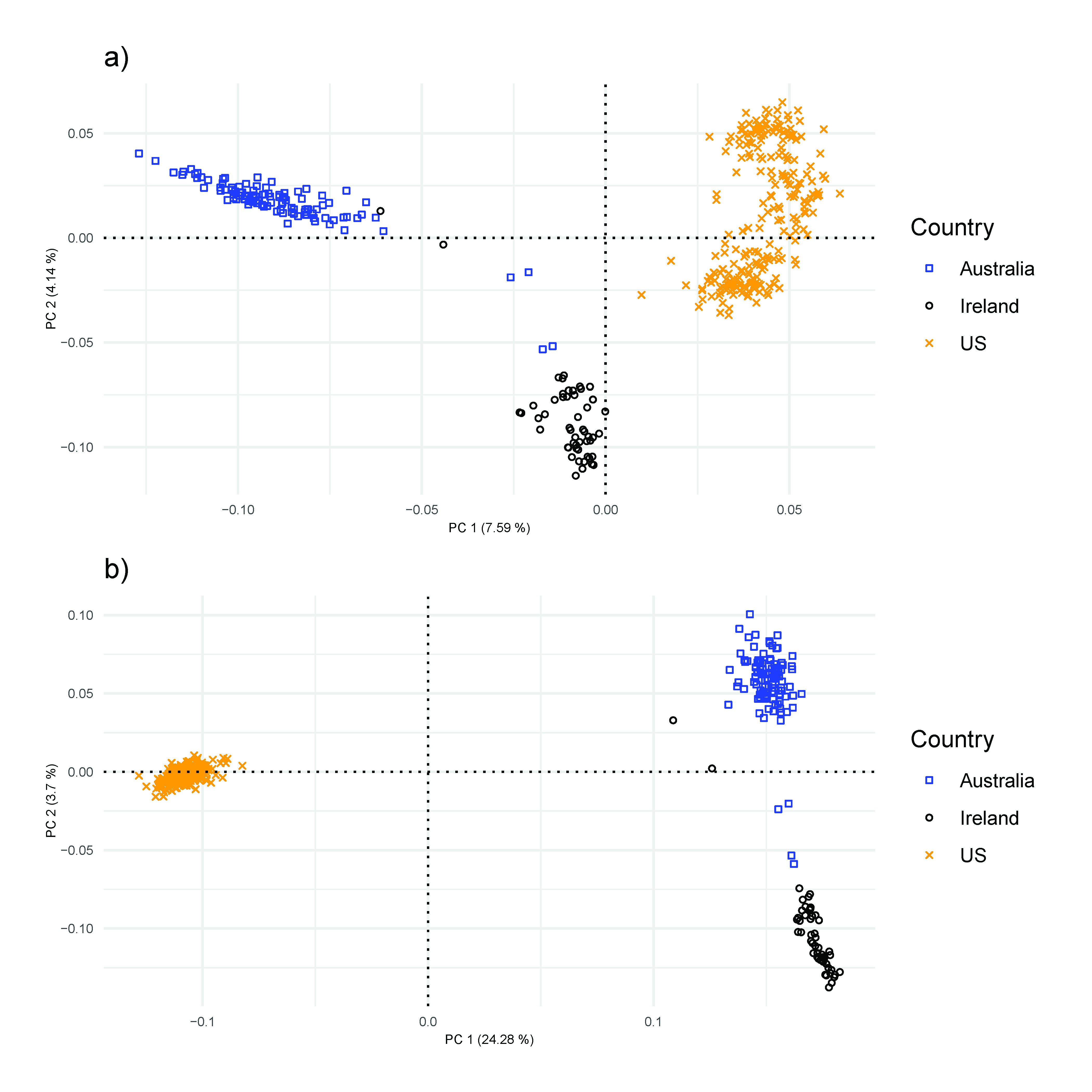

Supplement: Supplementary file 9 — Supplementary Material 9. [file 12711_2025_1027_MOESM9_ESM.jpg]

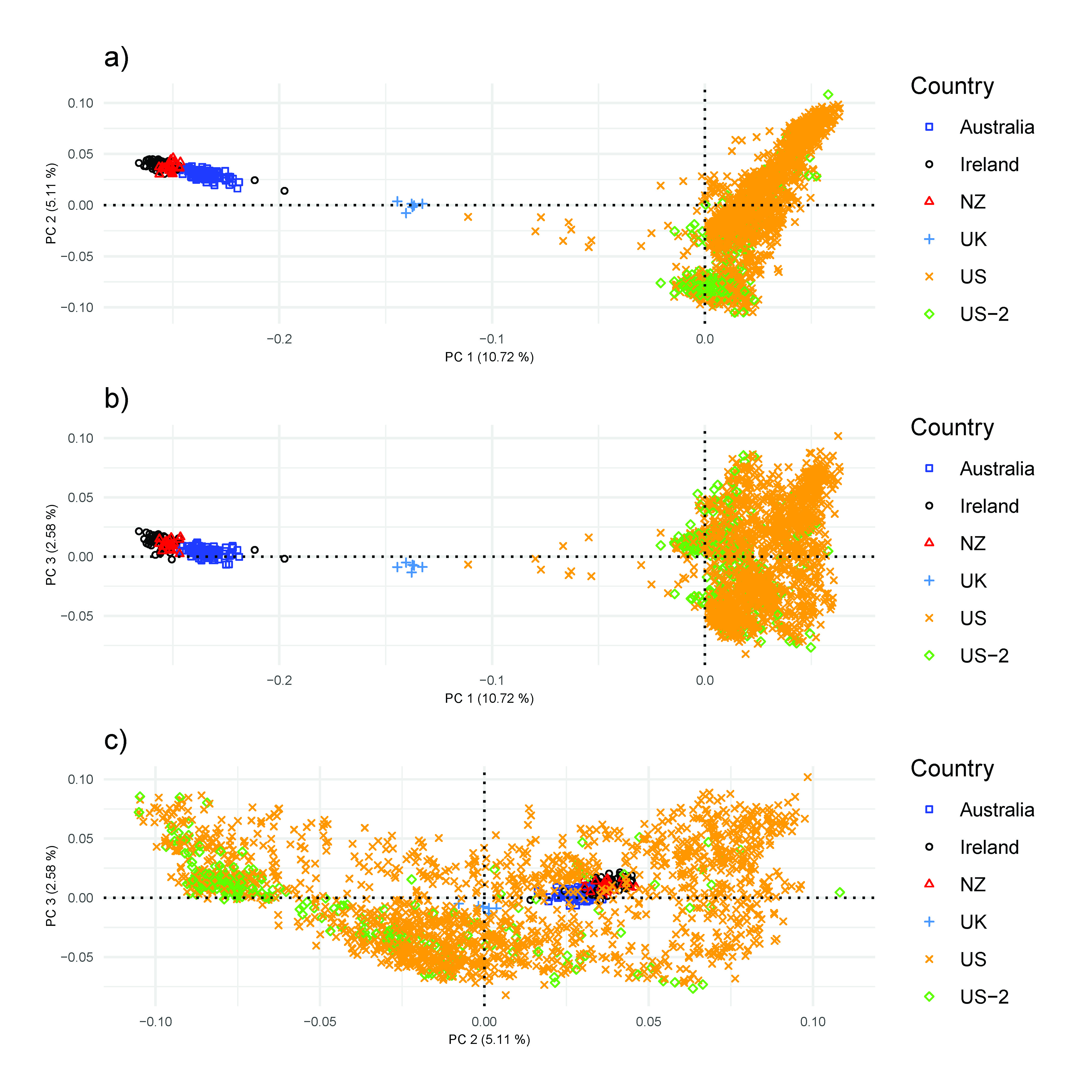

Supplement: Supplementary file 10 — Supplementary Material 10. [file 12711_2025_1027_MOESM10_ESM.jpg]
